# SLC4A9 deficiency is involved in Sjögren’s syndrome and enamel defects through RPS27-triggered ribosomal stress

**DOI:** 10.1038/s12276-026-01867-0

**Published:** 2026-10-09

**Authors:** Wenyan Ruan, Jing Dong, Yali Xu, Yanli Zhang, Yulong Zhu, Songya Mao, Yuan Liu, Xiaohong Duan

**Affiliations:** 1 State Key Laboratory of Oral & Maxillofacial Reconstruction and Regeneration, National Clinical Research Center for Oral Diseases, Shaanxi Key Laboratory of Stomatology, Department of Oral Biology & Clinic of Oral Rare Diseases and Genetic Diseases, School of Stomatology, the Fourth Military Medical University, Xi’an, China; 2 Department of Oral Pathology, School of Stomatology, the Fourth Military Medical University, Xi’an, China

**Keywords:** Medical research, Immunological disorders

## Abstract

Xerostomia, a salivary gland disorder affecting up to 30% of adults, compromises oral homeostasis by reducing salivary flow and buffering capacity. Amelogenesis imperfecta is a hereditary condition characterized by defective enamel formation and mineralization, with a prevalence ranging from 1 in 700 to 1 in 14,000 children worldwide. Although xerostomia has been associated with increased susceptibility to dental caries and subsequent enamel damage, its relationship with amelogenesis imperfecta remains unclear, and no shared molecular pathway has been causally linked to both hyposalivation and enamel malformation. Notably, several Cl^−^/HCO_3_^−^ exchangers regulate ion transport and pH homeostasis, thereby influencing salivary secretion as well as enamel maturation and mineralization, raising the possibility that these transporters may serve as a common molecular link between xerostomia and amelogenesis imperfecta. Here, we report the identification of SLC4A9, a Na^+^-dependent Cl^−^/HCO_3_^−^ exchanger whose dysfunction simultaneously disrupts salivary secretion and enamel mineralization. Genetic analyses identified variations in the *SLC4A9* gene, including missense mutations in three cases of amelogenesis imperfecta and copy number variations with reduced SLC4A9 expression in a patient with Sjögren’s syndrome. *Slc4a9* knockout mice exhibited dual pathological phenotypes, including salivary impairment with reduced secretion, female-predominant glandular inflammation and autoantibody production, as well as enamel malformation with delayed ameloblast differentiation. Mechanistically, SLC4A9 deficiency disrupted Cl^−^/pH homeostasis, leading to RPS27 upregulation and ribosomal stress, which in turn activated p53-dependent cell-cycle arrest and apoptosis in both glandular and enamel lineages. Treatment with the MDM2 antagonist Nutlin-3a reduced salivary inflammation, supporting the therapeutic potential of this pathway. Collectively, these findings suggest that xerostomia and amelogenesis imperfecta represent overlapping phenotypes driven by variations in the same gene and a shared mechanism of ribosomal stress-associated apoptosis, despite occurring in distinct tissues. This work highlights a unified diagnostic target and potential therapeutic strategy for restoring both salivary function and enamel integrity.

## SLC4A9 Alterations Connect Salivary Dysfunction and Enamel Defects

Sjögren’s syndrome is a chronic autoimmune disease characterized by dry mouth and eyes, with salivary gland dysfunction. This study explores the role of the *SLC4A9* gene, a Na^+^ -dependent Cl^−^/HCO3^−^ exchanger, in Sjögren’s syndrome pathogenesis and enamel defects. Researchers used genetic analysis and CRISPR/Cas9 to create *Slc4a9* knockout mice, revealing impaired salivary secretion and enamel mineralization. The study found that *Slc4a9* deficiency disrupts ion transport and pH regulation, leading to salivary gland inflammation and enamel hypoplasia. These findings suggest that SLC4A9 is essential for ionic homeostasis in salivary glands and developing teeth and may represent a potential therapeutic target for Sjögren’s syndrome and enamel defects. Future research could explore targeted therapies to restore ion balance and improve glandular and dental health in patients with Sjögren’s syndrome or amelogenesis imperfecta.

This summary was initially drafted using artificial intelligence, then revised and fact-checked by the author.

## Introduction

Sjögren’s syndrome is a chronic autoimmune disease characterized by dry mouth and/or eyes. The salivary and lacrimal glands affected exhibit lymphocyte infiltration with concomitant seropositivity for specific autoantibodies, such as anti-Ro/SSA and anti-La/SSB antibodies^1–3^. In the past few years, the etiology of Sjögren’s syndrome has been revealed from essential factors including virus infection, autoreactive T cells, B cell hyper-reactivity, autoantibodies, apoptosis of glandular epithelial cells and disrupted homeostasis of exocrine glands^4^. A few reports have also focused on the genetic background of Sjögren’s syndrome, variants in genes such as *IRF5*, *STAT4* and *FAM167A-BLK*, have been reported to be associated with Sjögren’s syndrome^5–7^.

Saliva secretion is primarily driven by transepithelial Cl^−^ transport. Cl^−^ uptake mainly occurs predominantly in acinar cells, generating a high intracellular Cl^−^ concentration — a prerequisite for adequate saliva production^8^. Dysregulation of Cl^−^/HCO_3_^−^ exchangers has been observed in the salivary glands of patients with Sjögren’s syndrome^9^. SLC4A9 belongs to the solute carrier family 4 (SLC4) and functions as a Na^+^-dependent Cl^−^/HCO_3_^−^ exchanger. It is highly expressed in the mouse kidney and submandibular gland (SMG), and *Slc4a9*^*−/−*^ mice exhibit a partial reduction in saliva secretion^10,11^. However, direct evidence linking Cl^−^/HCO_3_^−^ exchangers, specifically SLC4A9, to the pathogenesis of Sjögren’s syndrome remains lacking.

Cl^−^/HCO_3_^−^ exchangers such as SLC4A2 were also found in ameloblasts and might affect pH homeostasis during enamel formation. The salivary glands and teeth develop through continuous epithelial–mesenchymal interactions and share a common ectodermal origin^12^. Whether certain Cl^−^/HCO_3_^−^ exchangers could simultaneously regulate saliva secretion and enamel mineralization remains an open question. Moreover, enamel defects such as hypomineralization and discoloration observed in patients with Sjögren’s syndrome are typically attributed to salivary dysfunction^13–15^. However, the extent to which these defects stem primarily from secondary hyposalivation or the direct involvement of Cl^−^/HCO_3_^−^ exchangers in enamel mineralization is yet to be determined.

Sjögren’s syndrome has been extensively characterized as an autoimmune-mediated disease, and immunological studies have provided important insights into its pathogenesis and therapeutic strategies. However, current treatments targeting B cell depletion or inflammatory pathways show limited efficacy in restoring exocrine function^2^, suggesting that additional mechanisms beyond immune dysregulation may contribute to glandular dysfunction. In this context, disruption of ion transport processes, particularly Cl^−^/HCO_3_^−^ exchange, may represent an underexplored factor influencing salivary secretion. Notably, *Slc4a9* deficiency in mice impairs ionic homeostasis in acinar cells, leading to hyposalivation, and may further compromise the bicarbonate-dependent pH regulation required for enamel remineralization. Building on these observations, we propose that ion transport imbalance drives cross-tissue dysfunction linking salivary-gland impairment with enamel defects, thereby providing a unifying framework for understanding Sjögren’s syndrome and its association with enamel abnormalities.

## Materials and methods

### Patient recruitment and clinical examination

Three cases of amelogenesis imperfecta from two Chinese families were recruited from our Clinic of Oral Rare Diseases and Genetic Diseases. Clinical examination and cone-beam computed tomography were performed on all affected individuals to assess enamel and dentin integrity. Cases 1 and 2 were siblings presenting similar enamel abnormalities and reported a maternal family history. Case 3 was a sporadic amelogenesis imperfecta case with unknown family history. All amelogenesis imperfecta cases, their family members, and one patient with Sjögren’s syndrome recruited from our clinic underwent genetic analysis after providing written informed consent. In addition, de-identified archival formalin-fixed paraffin-embedded labial gland specimens were collected from our hospital for histological and immunohistological analysis. The samples were derived from 5 Sjögren’s syndrome patients and 6 control subjects. The patients with Sjögren’s syndrome were definitively diagnosed based on clinical, serological and histopathological criteria, whereas the control individuals presented with dry-mouth symptoms but lacked specific autoantibodies or histological evidence of Sjögren’s syndrome. This study was approved by the Institutional Review Board of the Fourth Military Medical University (Approval No. IRB-REV-2022182).

### Genetic and bioinformatic analyses

Peripheral blood samples were collected from all participating patients. Genomic DNA was extracted using the QIAamp DNA Blood Mini Kit (QIAGEN) according to the manufacturer’s instructions. Whole-exome sequencing was performed on the three affected individuals from Family 1 and the sporadic case from Family 2 using the BGISEQ-500 platform. Whole-genome sequencing was performed on patients with Sjögren’s syndrome using DNBSEQ-T7 platforms (PE150). Reads were aligned to GRCh38. Whole-exome sequencing variant calling focused on non-synonymous, splice-site and coding indel variants. Candidates were prioritized by minor allele frequency and predictive algorithms (SIFT, PolyPhen-2, MutationTaster, CADD) within known amelogenesis imperfecta-associated genes, followed by pedigree segregation analysis. Validation was conducted via PCR and Sanger sequencing. Missense variants were evaluated using conservation analysis, SWISS-MODEL and PyMOL 1.7. Whole-genome sequencing-related structural variations were detected using BreakDancer (v1.4.5), and CNVs were analysed using CNVnator (v0.4.1). Functional annotation was conducted with VEP (release 103).

### Animals

All animal procedures were approved by the Ethics Committee of the School of Stomatology, the Fourth Military Medical University(Approval No. 2017-015). *Slc4a9* knockout mice were generated via CRISPR/Cas9. The targeting site spanned exons 3–14 of the mouse *Slc4a9* gene (Ensembl: ENSMUSG00000024485) on chromosome 18. Single-guide RNA (sgRNA) expression vectors were constructed using a Church Lab gRNA cloning vector by Gibson assembly. *Slc4a9*-specific sgRNA and Cas9 mRNA, synthesized with the mMESSAGE mMACHINE T7 Kit (Life Technologies), were co-injected into C57BL/6 fertilized eggs to generate founders (Cyagen, China). Founder and F1 mice were genotyped by PCR and sequencing using the following primers: *Slc4a9*-Forward: 5’-GAGTGCTCCCCATGTGCCTACC-3’, *Slc4a9*-Wt/He-Forward: 5’-CCTCATGCGCTGTAGTTACAGACGTG-3’ and *Slc4a9*-Reverse: 5’-GGTTCAGACCTGCCTCAGACTGG-3’. Genotyping results were interpreted as follows: a single 433-bp product indicated wild type (WT) mice, a 560-bp product indicated *Slc4a9*^*−*^^*/*^^*−*^ mice, and both products indicated *Slc4a9*^*+/*^^*−*^mice. 4- and 6-month-old *Slc4a9*^*−/−*^ and WT littermates received intraperitoneal injections of Nutlin-3a (20 mg/kg in DMSO; TargetMol) or vehicle (normal saline) every 2 days for 1 month. Mucin accumulation and histological changes in the SMGs were assessed at the endpoint.

### Tissue fixation, embedding and sectioning

Tissue samples from various developmental and experimental conditions were collected and processed. These included heads from embryonic day 16.5 (E16.5), E18.5, and postnatal day 1 (P1) mice; SMGs from mice aged 3, 6, 10 and ≥ 12 months; and SMG from 5- and 7-month-old mice treated with nutlin-3a. All samples were fixed in 4% paraformaldehyde (48 h for heads, 24 h for SMGs). Following fixation, samples were dehydrated through a graded ethanol series, cleared in xylene and embedded in paraffin. Serial sections (4 μm) were obtained using a rotary microtome (HistoCore BIOCUT, Leica). Separately, maxillae from adult mice (≥ 3 months) were fixed in 4% paraformaldehyde, dehydrated, cleared and embedded in methyl methacrylate resin. Sections of these hard tissues were cut using a hard-tissue microtome.

### Haematoxylin and eosin staining

Sections from embryonic (E16.5 and E18.5) and postnatal P1 mouse heads, as well as from SMGs of 3-, 5- (treated with nutlin-3a), 6-, 7- (treated with nutlin-3a), 10- and ≥ 12-month-old mice, were deparaffinized in xylene and rehydrated through a graded ethanol series. Haematoxylin and eosin (H&E) staining was performed according to standard protocols using a commercial kit (Servicebio, Wuhan, China). After dehydration and clearing, slides were mounted with resinous medium and examined under a light microscope for morphological assessment.

### Periodic acid-Schiff and periodic acid-Schiff-diastase staining

Following deparaffinization and rehydration, paraffin sections of SMGs from 3-month-old and Nutlin-3a-treated mice (aged 5 and 7 months) were processed for periodic acid-Schiff-diastase (PAS-D) staining. These sections were digested with a 1% diastase solution at room temperature for 60 min prior to oxidation. As a control, adjacent sections designated for standard PAS staining were incubated in distilled water (without diastase) under the same conditions for the same duration. All subsequent steps were identical for both groups. Sections were oxidized in periodic acid solution for 15 min at room temperature, rinsed with tap water, and washed twice in distilled water. Samples were then incubated with Schiff reagent for 30 min in the dark, followed by a 5-min rinse under running tap water. Nuclei were counterstained with haematoxylin for 30 s, differentiated briefly in acid-differentiation solution, and blued in running water for 10–15 min. Finally, sections were dehydrated through a graded ethanol series, cleared in xylene and mounted with neutral gum for microscopic examination.

### Immunofluorescence and immunohistochemical staining

For immunofluorescence, cells on coverslips were fixed with 4% paraformaldehyde, permeabilized with 0.2% triton X-100 and blocked with 3% BSA. Tissue sections were deparaffinized, rehydrated, subjected to antigen retrieval in sodium citrate buffer (95–100 °C) and blocked with 5% BSA. All samples were incubated overnight at 4 °C with primary antibodies diluted in blocking buffer, including anti-RPS27 (1:200, Signalway Antibody), anti-B23 (1:100, Santa Cruz Biotechnology), anti-RPL7A (1:200, Proteintech), anti-SLC4A9 (1:300, Abcam), anti-SLC4A2 (1:100, Santa Cruz Biotechnology) and anti-AMELX (1:100, Santa Cruz Biotechnology). After washing, samples were incubated with fluorophore-conjugated goat anti-rabbit or goat anti-mouse secondary antibodies (CWBIO; 1:100–1:200) at 37 °C for 1 h. Nuclei were stained with DAPI, and images were acquired on a laser-scanning confocal microscope (Nikon). For immunohistochemistry, tissue sections were processed similarly through deparaffinization, rehydration, antigen retrieval and blocking. Sections were incubated overnight at 4 °C with primary antibodies against RPS27 (1:200, Signalway Antibody), MUC10 (1:200, Everest Biotech), MUC3A (1:50, Santa Cruz Biotechnology) or SLC4A9 (1:300, Affinity). After incubation with a secondary antibody, signal was developed with diaminobenzidine, counterstained with haematoxylin, dehydrated and mounted for observation under a light microscope.

### Transmission electron microscopy

Following anaesthesia, mice were euthanized and SMG tissues were rapidly excised. Fresh tissues were immediately immersed in a 2.5% glutaraldehyde fixative and diced into cubes smaller than 1 mm^3^. Tissues were then fixed in 2.5% glutaraldehyde for 24 h. Subsequently, samples were dehydrated through a graded ethanol series (50%, 70%, 80% and 90%) for 15–20 min per step, followed by a transition to 90% ethanol:90% acetone (1:1), 90% acetone (4 °C condition) and finally three changes of 100% acetone at room temperature (15–20 min each). Infiltration was performed at room temperature using a 2:1 mixture of pure acetone and embedding resin for 4 h, followed by a 1:2 mixture overnight. Samples were then infiltrated with pure embedding resin at 37 °C for 3 h and polymerization was carried out at 37 °C overnight, 45 °C for 12 h and 60 °C for 24 h. Ultrathin sections (50–60 nm) were cut using an ultramicrotome and stained with 3% uranyl acetate and lead citrate before observation under a transmission electron microscope.

### Micro-computed tomography scanning and analysis

Heads from adult mice (≥ 3months) of the indicated genotypes (WT, *Slc4a9*^*+/*^^*−*^ and *Slc4a9*^*−/−*^) were collected after euthanasia by cervical dislocation, stripped of soft tissue and fixed in 4% paraformaldehyde. Three-dimensional tooth microarchitecture was assessed using a high-resolution micro-CT system. Scans were performed at 80 kV, 500 μA and an exposure time of 500 ms. Three-dimensional reconstructions were generated with RadiAntViewer software. Each group included three mice (*n* = 3).

### Scanning electron microscopy analysis of enamel

Hard-tissue sections (contained molars and incisors) were etched with 35% phosphoric acid for 30 s. Subsequently, they underwent ultrasonic cleaning in absolute ethanol and de-ionized water (10 min each) to remove debris and grease. After air-drying, the samples were sputter-coated with gold and imaged using a Hitachi S-4800 field-emission scanning electron microscope.

### Saliva collection and analysis

Mice were anaesthetized via intraperitoneal injection of pentobarbital sodium (40 mg/kg) and immobilized on a surgical platform. Stimulated saliva secretion was induced by intraperitoneal administration of pilocarpine (0.5 mg/kg). After 5 min, pre-weighed sterile cotton balls were placed in the buccal pouch, and saliva was collected continuously for 30 min. The cotton balls containing saliva were then transferred into pre-weighed microcentrifuge tubes and weighed on a precision balance (0.1 mg resolution). Saliva flow was calculated as the weight difference before and after collection. To account for body size variation, secretion rates were normalized to body weight. The absolute secretion rate (mg/min) was divided by the body weight (g) of each mouse to obtain the normalized secretion rate (mg/min/g). All statistical comparisons were based on normalized secretion rates.

### Enzyme-linked immunosorbent assay

Serum levels of anti-SSB, anti-SSA and anti-ANA antibodies were assessed. Blood was obtained via the retro-orbital plexus from mice of both sexes, encompassing different genotypes and age groups (3, 6, 10 and ≥ 12 months), in addition to 5- and 7-month-old Nutlin-3a-treated *Slc4a9*^*−/−*^ and WT controls. Samples were centrifuged at 8,000 × *g* for 10 min at 4 °C. The serum was aliquoted and stored at −80 °C. SSA, SSB and ANA concentrations were quantified using commercial mouse enzyme-linked immunosorbent assay (ELISA) kits (MEIMIAN) following the manufacturer’s instructions.

### Cell culture and treatment

Primary tooth germ cells were isolated from P1 mouse maxillary first molars under aseptic conditions. Tissues were washed with PBS and digested with collagenase type I (1 mg/ml) for 40 min at 37 °C. After centrifugation, cells were resuspended in DMEM supplemented with 10% FBS and 1% penicillin/streptomycin and seeded in 12-well plates.

Primary SMG cells were isolated from P4 mice. Following PBS washing, tissues were digested with collagenase type II (1 mg/ml) for 40 min at 37 °C. Cells were collected by centrifugation and cultured in DMEM containing 10% FBS, 1% penicillin/streptomycin, 0.1 μg/ml EGF, 5 μg/ml insulin, 5 μg/ml transferrin and 0.4 μg/ml hydrocortisone in six-well plates. Both primary cell types were incubated at 37 °C under 5% CO_2_ and passaged once prior to experimentation.

LS8 cells (an SV40-immortalized mouse ameloblast-like cell line^16^) were maintained in high-glucose DMEM supplemented with 10% FBS, 100 U/ml penicillin and 100 μg/ml streptomycin at 37 °C with 5% CO_2_. Cells were transduced with lentiviral particles carrying WT *SLC4A9* (WT-*SLC4A9*), mutant *SLC4A9* (R300L or R115Q) or mCherry control. After 72 h, stable polyclonal populations were selected with 2 μg/ml puromycin for 2 weeks, yielding the following cell lines: *SLC4A9*-R300L, *SLC4A9*-R115Q, WT-*SLC4A9* and mCherry control.

### Induction of mineralization in cells and alizarin red staining

Primary tooth germ cells seeded at a density of 5 × 10^3^/well, 1 × 10^4^/well, 2.5 × 10^4^/well, 5 × 10^4^/well in 24-well plates. After 24 h, the mineralization induction medium containing 10 mM sodium β-glycerophosphate, 1 × 10^−7 ^M dexamethasone and 50 mg/ml ascorbate acid was added. The cells were cultured in osteogenic and control medium in a 24-well plate for 7, 14 and 21 days and 1% Alizarin red staining kit (Solarbio, China) was used to identify the formation of calcified nodules.

### Intracellular Cl^−^ and pH measurements

Primary cells and transduced LS8 cells were seeded on glass-bottomed dishes at a density of 5 × 10^3^ cells/well for live-cell imaging. To measure intracellular Cl^−^, cells were loaded with 5 mM MQAE for 60 min; for pH measurements, cells were loaded with 2 μM BCECF-AM for 15 min. Fluorescence imaging was performed on a Leica DM6000B microscope under identical exposure settings for all groups. MQAE was excited at 340 nm and emission was collected at 510 nm; BCECF was excited alternately at 440 nm and 490 nm, with emission collected at 530 nm. Changes in blue (MQAE) and green (BCECF) fluorescence intensities were recorded. During imaging, cells were perfused with the following bath solutions (in mM): Buffer 1 (containing Cl^−^): 4.3 KCl, 120 NaCl, 25 NaHCO_3_, 5 glucose, 10 HEPES, 1 CaCl_2_, 1 MgCl_2_, pH 7.4. Buffer 2 (free from Cl^−^): 4.3 potassium gluconate, 120 sodium gluconate, 25 NaHCO_3_, 5 glucose, 10 HEPES, 1 CaCl_2_, 1 MgCl_2_, pH 7.4.

### Western blot analysis

Total protein was extracted using RIPA buffer (50 mM Tris-HCl pH 7.4, 150 mM NaCl, 1% NP-40, 1 mM EDTA) supplemented with protease inhibitors (1 mM PMSF, 1 µg/ml leupeptin, 1 µg/ml aprotinin). For nuclear and cytoplasmic fractionation, the Nuclear and Cytoplasmic Protein Extraction Kit (PK10014, Proteintech) was used according to the manufacturer’s instructions, with protease inhibitor cocktail (×100) added to all pre-chilled buffers. Briefly, cells or tissues were homogenized in ice-cold cytoplasmic extraction reagent A, followed by centrifugation at 6500 × *g* to collect the cytoplasmic supernatant. The nuclear pellet was washed twice with nuclear wash reagents, resuspended in nuclear extraction reagent B and centrifuged at 16,000 × *g* to obtain the nuclear fraction. Protein concentration was determined using a BCA assay kit (Cwbio). Equal amounts of protein (50 µg per lane) were separated on 10% or 12% SDS-PAGE gels and transferred to PVDF membranes (Millipore). Membranes were blocked with 5% non-fat milk in TBST for 1 h at room temperature, then incubated overnight at 4 °C with primary antibodies diluted in blocking buffer: anti-SLC4A9 (1:500, Thermo Fisher), anti-RPS27 (1:300, Signalway Antibody), anti-MDM2 (1:3000, Proteintech), anti-p53 (1:10000, Proteintech), anti-AMELX (1:200, Santa Cruz), anti-DSPP (1:200, Abmart), anti-DMP1 (1:200, Abmart) and anti-GAPDH (1:7000, ZEN-BIOSCIENCE). After three washes in TBST, membranes were incubated for 1 h at room temperature with fluorophore-conjugated goat anti-mouse or anti-rabbit secondary antibodies (1:5000, LI-COR). Signals were detected using an Odyssey CLX imaging system (LI-COR), and quantification was performed using Image Studio software.

### RNA-sequencing analysis

Whole-transcriptome sequencing was accomplished by BGI (Wuhan, China). WT, *Slc4a9*^*+/*^^*−*^ and *Slc4a9*^*−*^^*/*^^*−*^ mouse teeth tissue of P1 and SMG tissue of 3 months were quickly separated, and the samples were pretreated and prepared in accordance with the whole transcriptome sequencing standards. Samples were sequenced on the DNBSEQ platform. Bowtie2 software (v2.2.5) was used to align clean reads to reference gene sequence, and RSEM (v1.2.8) was used to calculate the expression levels of genes and transcripts. Differential expression analysis was performed using DESeq2 (v1.4.5) with a Q value  ≤  0.05 considered to indicate significantly differential expression. Heatmaps from significantly differential gene expression were generated using pheatmap.

### Single-cell RNA sequencing

Single-cell RNA sequencing was performed using 10 × Genomics Single Cell Platform. We separated the first maxillary molar tooth germ from P1. Sample preparation, single cell capture, reverse transcription, cDNA amplification, library construction, RNA sequence and data analysis were performed by Biomarker Technologies Co., Ltd. (Beijing, China). For the raw sequence data, Cell Ranger software was used to obtain the gene-expression matrix. Subsequent data analysis including standardization, cell subpopulation, principal component analysis, t-distributed stochastic neighbour embedding, uniform manifold approximation and projection, cell annotation and marker gene screening were achieved using Seurat software. The “AddModuleScore” function in Seurat was applied for analysis of the cell-cycle score. Furthermore, we compared the differentiation trajectories of cell clusters using the Monocle 2 algorithm.

### Statistical analysis

Data were presented as mean ± SD. Comparisons between two independent experimental groups were performed using unpaired Student’s *t* test. Comparisons of each treatment group with its control were performed using Dunnettʼs *t* test. The difference was considered to be statistically significant when the *P* value was less than 0.05. Each experiment was repeated at least three times.

## Results

### Reduced SLC4A9 level and *SLC4A9* variants in patients with primary Sjögren’s syndrome

Five female patients with primary Sjögren’s syndrome, and six women with non-Sjögren’s syndrome sicca (controls) were included in the study. Inclusion criteria of Sjögren’s syndrome were based on American College of Rheumatology/European League Against Rheumatism^17^ (Supplementary Table 1). None of the patients had a history of head and neck radiation treatment, active hepatitis C infection, AIDS, sarcoidosis, amyloidosis, graft-versus-host disease or IgG4-related disease.

**Fig. 1:**
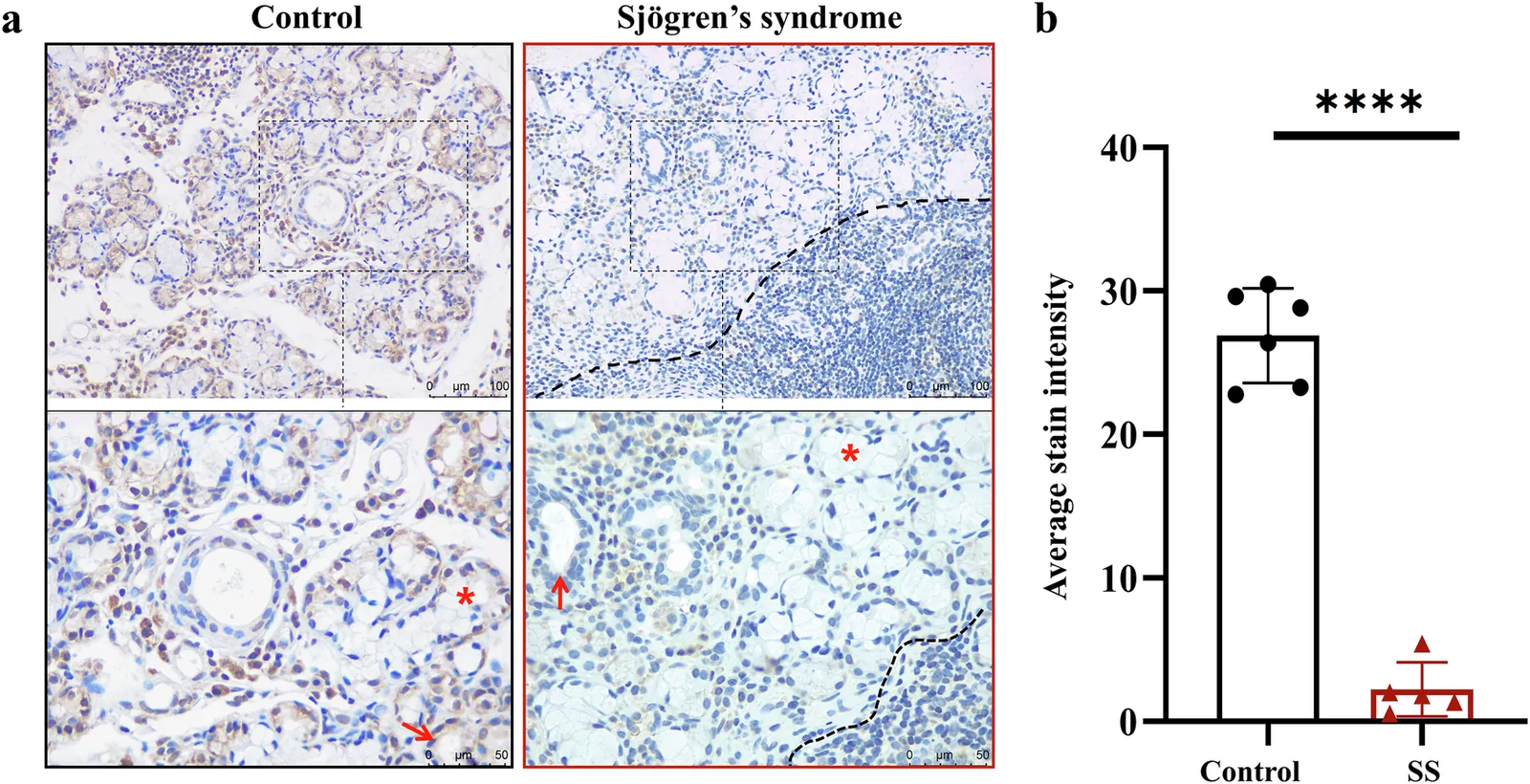
Reduced expression of SLC4A9 in patients with primary Sjögren’s syndrome. **a** Representative images of immunohistochemical staining of SLC4A9 in controls (*n* = 6) and individuals with primary Sjögren’s syndrome (SS) (*n* = 5). The black-dashed rectangular areas in the upper panels are shown at higher magnification than the lower panels. Objective magnification ×20. Positive signal: brown DAB signal; counterstain: haematoxylin/blue. Red arrow: ductal epithelium; red asterisk: acinar cells; black dashed line: the inflammatory region. Immunoreactivity of SLC4A9 is weakened in patients primary Sjögren’s syndrome. **b** Morphometric analysis of staining intensity of positive signal for SLC4A9 across the whole tissue section. Data are represented as individual points for each subject. Statistical significance was determined by *t* test: ****P < 0.0001.

Immunohistochemical analysis revealed distinct SLC4A9 expression patterns between the two cohorts. Notably, minor salivary gland specimens from control without Sjögren’s syndrome exhibited robust SLC4A9 immunoreactivity in ductal and acinar epithelial compartments (Fig. 1a). Conversely, patients with primary Sjögren’s syndrome demonstrated markedly reduced SLC4A9 expression levels in both glandular epithelial and infiltrating mononuclear cells, with only residual staining detectable (Fig. 1). These observations suggested a potential mechanism: whether the observed downregulation of SLC4A9 represents a secondary consequence of autoimmune processes or plays a primary role in the pathogenesis of Sjögren’s syndrome through its functions in epithelial homeostasis and immune modulation.

To assess whether patients with primary Sjögren’s syndrome may have *SLC4A9* mutations, we collected peripheral blood from one of the patients and extracted DNA for whole-genome sequencing analysis. A copy number variant (CNV) (chr5:140369102-140370800, a heterozygous deletion) was detected in *SLC4A9*, which is located in the intronic region and results in an uncertain effect on *SLC4A9* function. Bioinformatics analysis of ChIP-seq data from ENCODE cell lines revealed two constitutively occupied CCCTC-binding factor (CTCF) binding sites within the *SLC4A9* genomic locus, both localized within CNV deletion regions in patients with Sjögren’s syndrome (Supplementary Fig. 1). CTCF is an 11-zinc finger that functions as a transcriptional repressor or activator, as an insulator that can block the ability of enhancers to activate promoters, and mediates chromatin insulation and gene expression by binding 12- to 20-bp DNA motifs (CTCF binding sites) and altering the 3-dimensional chromatin structure^18,19^. This structural vulnerability suggests that the observed CNV-mediated loss of CTCF anchoring sites likely disrupts the native chromatin configuration of *SLC4A9*. These findings propose a possible pathogenic mechanism wherein SLC4A9 deficiency in Sjögren’s syndrome originates from insufficient functionality rather than secondary inflammatory effects.

### *Slc4a9* deficiency disrupts salivary-gland function, ultrastructure and mucin homeostasis

**Fig. 2:**
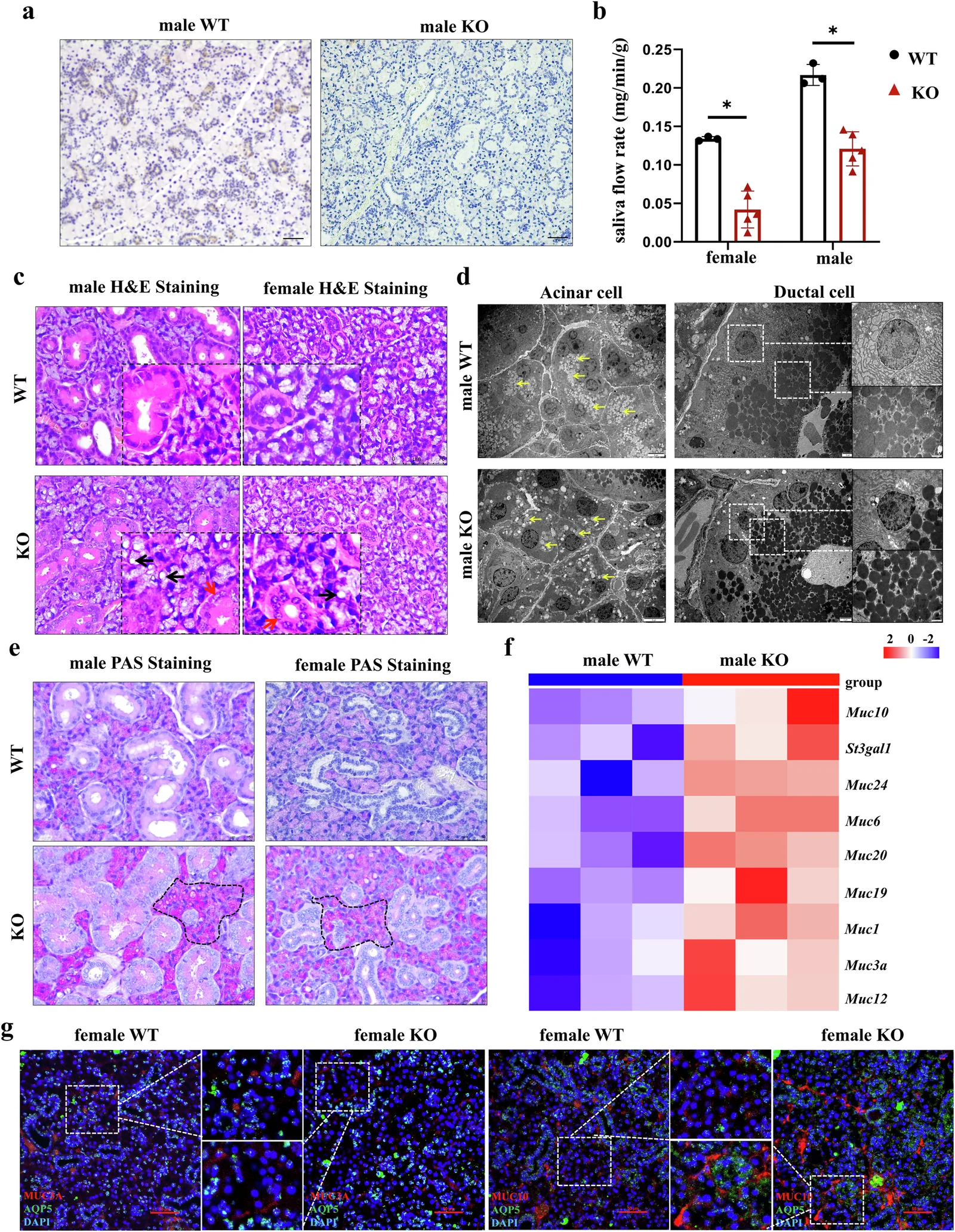
*Slc4a9* deficiency disrupts salivary gland function, ultrastructure and mucin homeostasis. **a** Spatial expression of SLC4A9 in submandibular glands (SMGs). Immunohistochemistry of SMG sections from wild type (WT) and homozygous knockout (KO) mice. SLC4A9 protein (brown DAB chromogen) predominantly localized to ductal cells, and nearly absent in homozygous knockout mice. Nuclei counterstained with haematoxylin (blue). **b** Functional assay of salivary flow in adult mice (WT: *n* = 3, KO: *n* = 5). Total saliva collected over 30 min and salivary volume was determined gravimetrically. Data are represented as individual points for each mouse. Statistical significance was determined by *t* test: *P < 0.05. Pilocarpine-stimulated saliva flow rates in adult mice revealed significant impairment in KO cohorts. **c** Histopathological alterations in SMGs. haematoxylin and eosin (H&E) staining of KO mice compared with WT littermates reveals pathological changes. Higher-magnification insets (within black dashed-line boxes) illustrate irregular nuclear contours, aberrant chromatin distribution and partial nuclear pyknosis in ductal epithelial cells (indicated by red arrows). Degenerative changes in the acinar compartment, including cytoplasmic vacuolation and reduced acinar size (marked by black arrows), are evident. Concurrently, ductal lumens display an accumulation of eosinophilic proteinaceous deposits. **d** Ultrastructural abnormalities by transmission electron microscopy. Left column: acinar structure composed of multiple acinar cells. Yellow arrows indicate secretory granules in acinar cells. Secretory granules in KO mice are scattered, reduced in number, and increased in size (versus WT). Right column: ductal structure. Areas outlined by white dotted lines represent the nuclei of ductal cells and intraductal secretions, respectively, with corresponding regions magnified in the black boxes. Ductal cells in KO mice show nuclear pyknosis and remodelling of secretory granules. **e** Enhanced mucin accumulation in acinar cells. Periodic acid-Schiff (PAS) staining demonstrated increased mucin density (dotted lines) in KO SMGs. **f** Transcriptional upregulation of mucin-related genes. Heatmap of differentially expressed mucins from SMG RNA-seq. Hierarchical clustering distinguishes WT (*n* = 3) and KO (*n* = 3) cohorts. Colour scale: Z-score normalized expression. **g** Immunofluorescence staining of MUC3A and MUC10 in SMG. AQP5 marks acini and ducts (green), MUC3A/MUC10 are shown in red and DAPI in blue. MUC3A and MUC10 showed limited co-localization with AQP5 and formed large punctate or cord-like aggregates around ducts and acinar peripheries. KO mice exhibited increased expression of both mucins, particularly MUC10, compared with WT mice.

To test this hypothesis and elucidate the functional role of SLC4A9, we generated *Slc4a9* knockout mice using CRISPR/Cas9-mediated targeting (Supplementary Fig. 2a–c). Immunohistochemical staining with anti-SLC4A9 monoclonal antibody confirmed ablation of transporter expression in SMG acini and ducts of homozygous knockout (*Slc4a9*^*−*^^*/−*^) mice compared with staining in WT mice (Fig. 2a). Functional assessment of salivary hypofunction was performed in both male and female *Slc4a9*^*−*^^*/−*^ and WT mice (≥ 3 months) under standardized conditions. Following intraperitoneal pilocarpine injection, *Slc4a9*^*−*^^*/−*^ mice exhibited a markedly reduced stimulated salivary flow rate (normalized to body weight) compared with controls, with decreases of approximately 78.6% in females and 50% in males (Fig. 2b). This secretory impairment magnitude aligns with prior reports of sodium-bicarbonate cotransporter dysfunction in exocrine epithelia^11^.

Comprehensive histopathological evaluation of *Slc4a9*^*−*^^*/−*^ mouse salivary glands revealed secretory dysfunction. Higher-magnification insets of H&E-stained sections revealed pathological alterations in *Slc4a9*^*−*^^*/−*^ glands compared with WT controls. Ductal epithelial cells exhibited irregular nuclear contours, aberrant chromatin distribution and partial nuclear pyknosis. Concurrently, ductal lumens displayed an accumulation of eosinophilic proteinaceous deposits. Moreover, the acinar compartment also exhibited degenerative changes, including vacuolation and atrophy (Fig. 2c). Transmission electron microscopy unveiled ultrastructural hallmarks of secretory collapse. Acinar cells from wild type mice exhibited typical morphology characterized by multiple acinar cells forming each acinus, with densely clustered, uniformly sized secretory granules in the cytoplasm. In contrast, acinar cells from *Slc4a9*^*−*^^*/−*^ mice showed a reduced number of secretory granules, which were notably enlarged (Fig. 2d). Ductal architecture displayed characteristic degenerative features, including nuclear pyknosis and secretory granule remodelling. Specifically, apical secretory granules exhibited a reduced size with increased packing density. Furthermore, the spatial distribution of secretory granules was altered — although WT controls showed apical clustering, *Slc4a9*^*−*^^*/−*^ mice displayed granule dispersion to basal regions and perinuclear areas (Fig. 2d).

Mucins, major components of saliva, are frequently altered in salivary-gland pathologies and serve as indicators of acinar-cell status. Histochemical and molecular analyses revealed pervasive mucin dysregulation in *Slc4a9*^*−*^^*/−*^ salivary glands. Regular PAS staining demonstrated increased mucin retention in both ductal and acinar compartments compared with WT controls (Fig. 2e). Consistently, transcriptomic analysis identified the upregulation of multiple mucin genes, including gel-forming mucins (*Muc6*, *Muc10* and *Muc19*) that contribute to the salivary mucus barrier, and membrane-associated mucins (*Muc1*, *Muc3a* and *Muc24*), which are involved in epithelial adhesion^20^ (Fig. 2f). MUC10 is considered a marker for the differentiation and maturation of serous acinar cells, and its aberrant expression is closely associated with disrupted acinar structure and impaired secretory function. Furthermore, considering that membrane-associated mucins such as MUC3A are critical for epithelial adhesion and tissue integrity, we assessed their expression alongside MUC10. To further validate these mucin changes at the protein level, we used AQP5 as a marker of the acinar and ductal systems and systematically examined the co-localization and expression patterns of MUC10 and MUC3A with AQP5. Immunofluorescence staining showed that AQP5 was mainly expressed in acinar cells and intercalated duct cells. Little co-localization was observed between AQP5 and either MUC10 or MUC3A; instead, MUC10 and MUC3A were predominantly distributed around the ducts or at the periphery of acinar cells. Notably, the residual mucin signals appeared as large punctate or cord-like aggregates, and the number of positive signals, particularly for MUC10, was increased in the *Slc4a9*^*−*^^*/−*^ SMGs (Fig. 2g). This upregulation occurred alongside concurrent suppression of glycoprotein-degrading enzymes (*Ugp2*) and enhancement of glycogen synthases (*Pygm, Gaa, Phka1*) (Supplementary Fig. 2d), suggesting aberrant glycoconjugate metabolism. This metabolic shift may underlie the observed structural disorganization, including acinar-cell atrophy, nuclear pyknosis and loss of secretory granule polarity. Collectively, *Slc4a9* deficiency drives salivary hyposecretion and architectural disruption, implicating intrinsic ion transport dysfunction as the primary pathology.

### *Slc4a9*^*−*^^*/−*^ mice developed Sjögren’s syndrome-like phenotypes

**Fig. 3:**
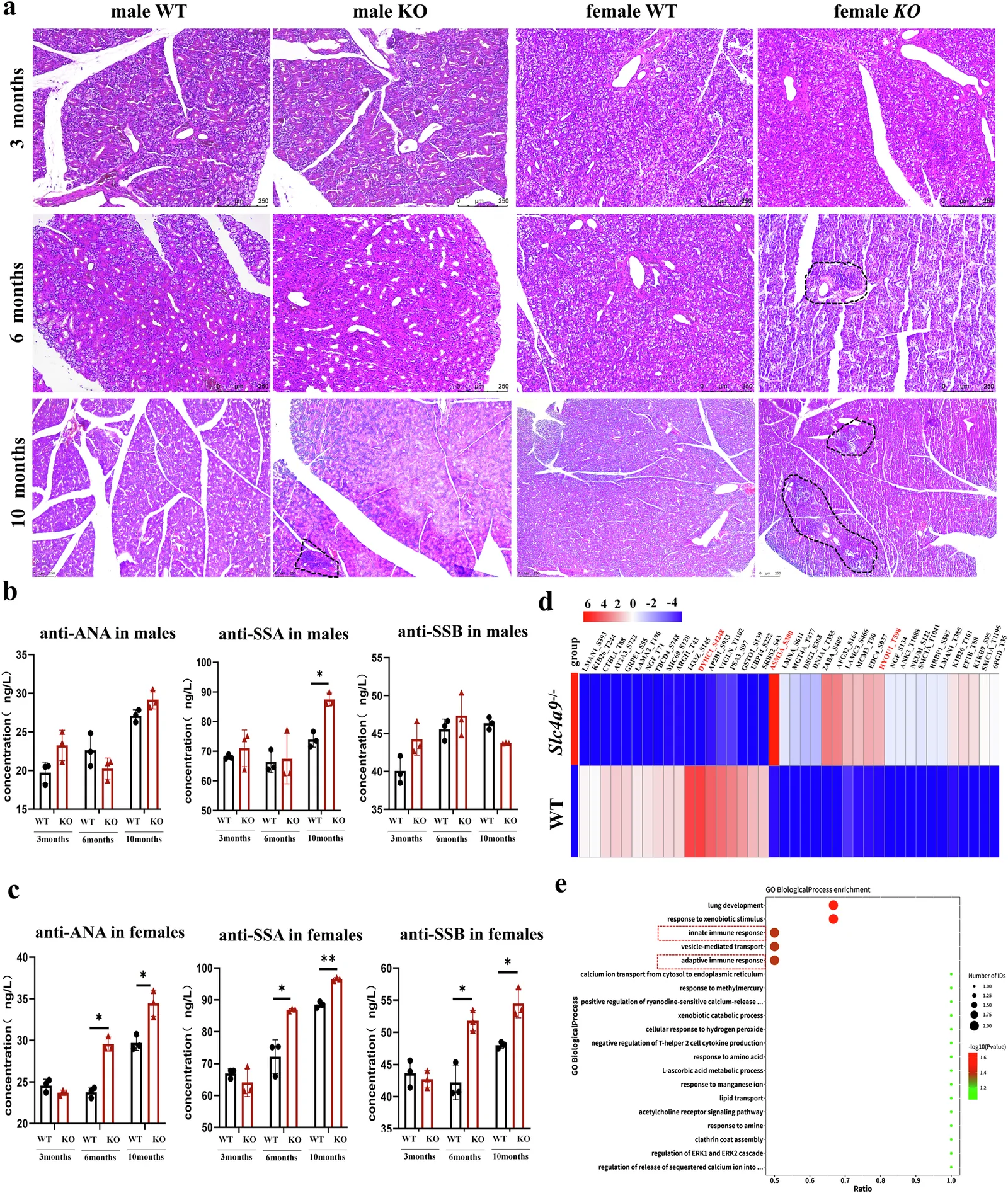
*Slc4a9* deficiency drives age-dependent Sjögren’s syndrome-like pathology and autoantibody production. **a** Lymphocytic infiltration in submandibular gland. H&E-stained submandibular gland sections from 3-, 6- and 10-month-old mice. Diffuse mononuclear cell infiltrates (demarcated by dashed outlines) were evident in *Slc4a9*^*−*^^*/−*^ mice, exhibiting age- and sex-dependent severity (female > male; 10 months > 6 months > 3 months). Wild type (WT) controls showed no pathology. **b** Male autoantibody profiles. Serum anti-SSA titres in 10-month-old *Slc4a9*^*−*^^*/−*^ male mice were significantly elevated versus age-matched WT. Data are represented as individual points for each mouse. Statistical significance was determined by *t* test: *P < 0.05. **c** Female autoantibody profiles. Female *Slc4a9*^*−*^^*/−*^ mice had elevated specific autoantibody levels at the age of 6 and 10 months compared with age-matched WT mice. Data are represented as individual points for each mouse. Statistical significance was determined by *t* test: *P < 0.05, **P < 0.01. **d** Differential modification signatures of salivary glycoproteins. Heatmap depicting expression profiles of 45 post-translational glycosylation sites across experimental groups. Expression levels (z-score range: −4 to 6; red = high, blue = low) revealed significant alterations in glycosylation sites of immunoregulatory molecules, e.g. ASM3A (S300), HYOU1 (T598) and DYHC1 (S4284). **e** Differential glycosylation of salivary proteins correlates with immune responses. Gene ontology enrichment analysis of biological processes revealed significant involvement in immune-related pathways, including innate/adaptive immune responses (shown as red frame), indicating their critical regulatory roles. KO, knockout.

Building on this intrinsic dysfunction, we further evaluated its long-term immunological consequences. Long-term pathological evaluation revealed progressive immune-mediated damage in *Slc4a9*^*−*^^*/−*^ salivary glands, exhibiting gender differences in patterns. Based on previous reports that the mouse model of Sjögren’s syndrome first manifests between 4 and 6 months of age^21^, we histologically assessed phenotypes at sequential timepoints (3, 6 and 10 months). Focal lymphocytic infiltration was evident in female knockout mice as early as 6 months of age and had expanded in both area and density by 10 months (Fig. 3a). In contrast, age-matched male knockouts displayed delayed onset of focal infiltration, which was not observed until 10 months and was less severe than in females. WT controls showed no structural or inflammatory abnormalities at any timepoint examined (Fig. 3a). Longitudinal serological profiling further revealed age-progressive autoantibody production in *Slc4a9*^*−*^^*/−*^ mice. Anti-nuclear antibodies (ANAs) are characteristic serological markers in multiple autoimmune diseases, detectable in 60% to 85% of patients with Sjögren’s syndrome and serving as a diagnostic hallmark^22^. Critically, anti-SSA/Ro and anti-SSB/La autoantibodies are primary serological criteria for Sjögren’s syndrome, formally integrated into the American–European Consensus Group (AECG) classification criteria as disease-defining tools^23^. Results showed that male knockouts displayed selective elevation of anti-SSA/Ro at 10 months (*P* < 0.005 versus WT), while maintaining WT-equivalent levels of ANAs and anti-SSB/La (Fig. 3b). By contrast, age-matched female knockouts exhibited significantly elevated titres of anti-ANA, anti-SSA/Ro and anti-SSB/La versus WT controls from 6 months onwards (*P* < 0.005 for all; Fig. 3c).

Although mild lymphocytic infiltration has been reported in WT C57BL/6 mice as early as 9 months of age^24,25^, no such infiltration was observed in 10-month-old WT mice in our cohort, whereas *Slc4a9*-deficient mice had already developed inflammatory changes by this age. To determine whether this immune phenotype reflected an accelerated disease process rather than a normal age-associated change, we further evaluated histopathological and serological profiles in aged (≥12 months) mice. H&E staining showed that lymphocytic infiltration became detectable in the salivary glands of both WT and knockout (KO) mice at this advanced age (Supplementary Fig. 3a). However, the infiltrated area was significantly greater in KO mice, particularly in females (Supplementary Fig. 3b). Consistently, serological analysis revealed significant increases in anti-ANA, anti-SSA/Ro and anti-SSB/La autoantibodies in aged female KO mice compared with age-matched WT controls (Supplementary Fig. 3c). Together, these findings indicate that *Slc4a9* deficiency accelerates the onset of salivary-gland inflammation and exacerbates its severity with ageing, especially in females.

Previous mouse models of Sjögren’s syndrome commonly develop lymphocytic infiltration, reduced salivary secretion, autoantibody production and salivary-gland cell apoptosis, with more severe phenotypes often observed in females^4^. Consistent with these features, our *Slc4a9*-deficient mice developed a Sjögren’s syndrome-like phenotype. In addition to inflammatory and functional abnormalities, these mice exhibited altered glycosylation and increased mucin levels. This is of particular relevance because patients with Sjögren’s syndrome also show aberrant glycosylation of salivary mucins, such as MUC5B and MUC7, which can affect salivary viscosity, mucosal clearance and hyposalivation^26^. Moreover, dysregulated IgG and IgA glycosylation may amplify autoantibody-mediated inflammation^27^. Thus, *Slc4a9* deficiency promotes a Sjögren’s syndrome-like salivary gland phenotype that may involve mucin dysregulation and aberrant glycosylation.

To investigate the underlying glycosylation alterations, we performed *Slc4a9*^*−*^^*/−*^ O-glycomic profiling of salivary-gland proteins. *Slc4a9*^*−*^^*/−*^ mice exhibited profound O-glycosylation remodelling versus WT, characterized by hyper-O-glycosylation at ASM3A_S300 (*P* = 0.0001) and HYOU1_T598, and hypo-O-glycosylation at DYHC1_S4248 (Supplementary Table 2, Fig. 3d). ASM3A functions as an innate immune checkpoint by suppressing TLR4/NFκB signalling and attenuating inflammatory cascades, thereby modulating immune homeostasis^28^. HYOU1 is mechanistically linked to endoplasmic reticulum (ER) stress and primary immunodeficiencies through dysregulated protein folding homeostasis, implicating its role in cellular stress responses. DYHC1 disrupts RNA-binding capacity within immunodeficiency 99 pathways, impairing mRNA processing fidelity and contributing to immune dysregulation^29^. Meanwhile, functional validation via gene ontology enrichment revealed these differentially glycosylated molecules are implicated in key pathological cascades (Fig. 3e), with significant involvement in immune-related pathways (e.g. innate/adaptive immune responses; highlighted by a red frame), indicating critical regulatory roles. Protein interaction analysis revealed direct binding between these glycosylated targets (Lmna, Ank3, Dsg2, Hyou1) and mucins (Supplementary Fig. 3d). Together, these results suggest that aberrant glycosylation programmes linked to mucin dysregulation may contribute to mucin aggregation and glycan-dependent lymphocyte recruitment, thereby exacerbating glandular inflammation and dysfunction in the Sjögren’s syndrome-like phenotype.

### *Slc4a9*^*−*^^*/−*^ mice present enamel hypoplasia

**Fig. 4:**
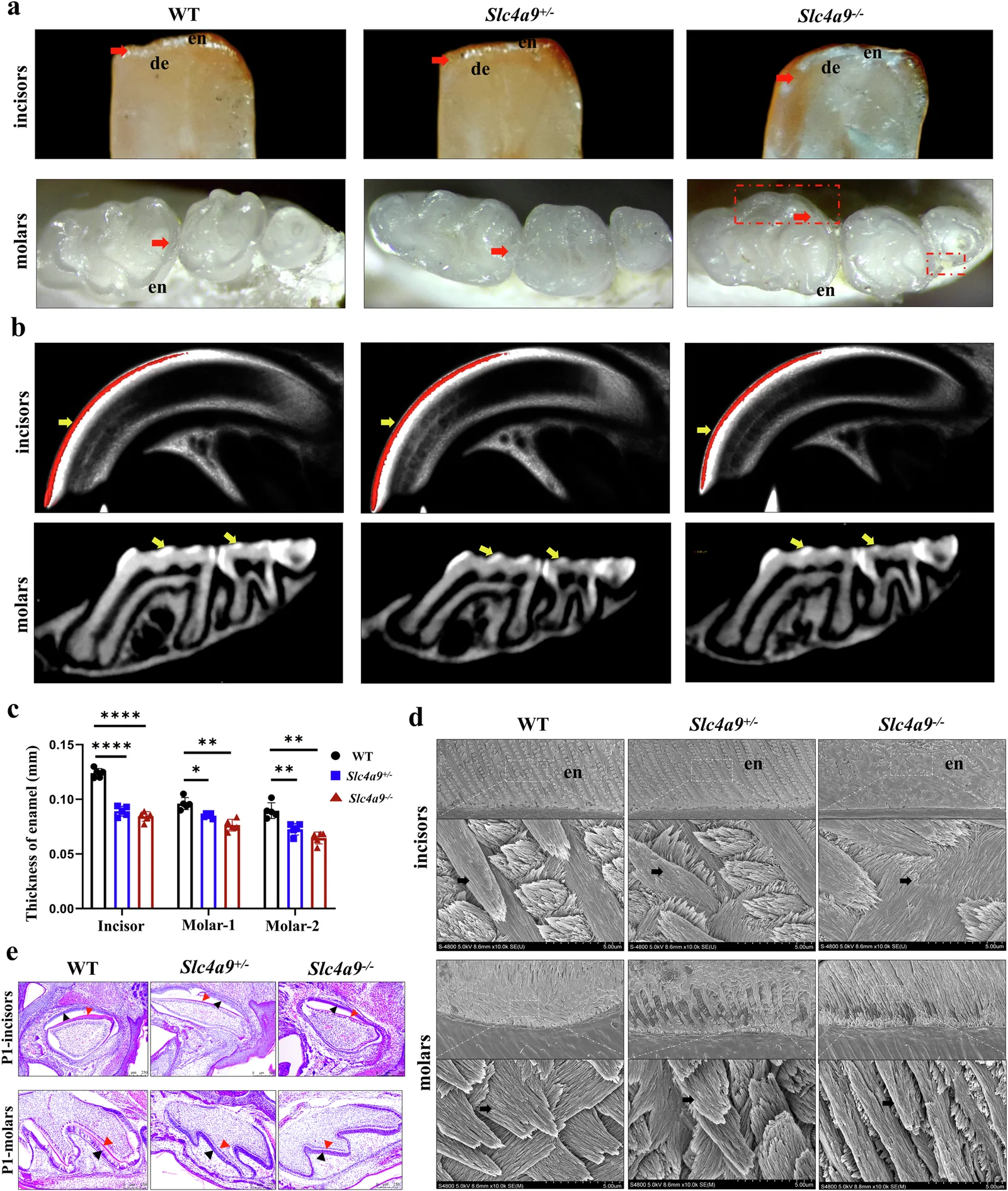
Enamel defects in *Slc4a9* knockout mice. **a** Macroscopic enamel defects. Maxillary incisors and molars from adult (≥ 3 months old) wild type (WT), *Slc4a9*^*+*^^*/−*^ and *Slc4a9*^*−*^^*/−*^ mice are shown. *Slc4a9*^*−*^^*/−*^ incisors exhibited chalky-white hypomineralized enamel with irregular wear, in contrast to the translucent, mineralized enamel in WT mice. *Slc4a9*^*−*^^*/−*^ molars showed enamel exfoliation at the cusps and cuspal defects, whereas WT molars retained an intact enamel surface. Red arrow: enamel layer. de, dentin; en, enamel. **b** Micro-CT analysis of enamel thickness. Sagittal images of incisors and coronal images of molars show reduced enamel thickness in *Slc4a9*^*−*^^*/−*^ mice compared with WT littermates. Yellow arrows indicate the labial enamel margin in incisors and the occlusal enamel in molars. **c** Quantification of enamel thickness by micro-CT. Enamel thickness was significantly reduced in *Slc4a9*^*−*^^*/−*^ mice compared with WT mice in both incisors and molars. Data are individual values for each mouse. Statistical significance was determined by *t* test: *P < 0.05, **P < 0.01, ****P < 0.0001. **d** Disrupted enamel ultrastructure. Scanning electron microscopy of maxillary incisors and first molars revealed disorganized enamel architecture in *Slc4a9*^*−*^^*/−*^ mice compared with WT controls. Boxed regions shown at higher magnification (bottom) demonstrate porous, loosely packed enamel rods (black arrows) in *Slc4a9*^*−*^^*/−*^ mice. En, enamel. **e** Impaired enamel formation. H&E-stained sections of the maxillary incisor and first molar at postnatal day 1 (P1) show that *Slc4a9*^*−*^^*/−*^ incisors and molars lacked detectable enamel deposition compared with WT controls. Red arrowhead: enamel matrix. Black arrowhead: ameloblasts.

Normal salivary flow is required for enamel remineralization by maintaining ionic homeostasis and buffering capacity. To assess whether SLC4A9 deficiency affects enamel integrity, we compared teeth from *Slc4a9*^*−*^^*/−*^ and WT mice. *Slc4a9*^*−*^^*/−*^ mice exhibited striking enamel defects, including cuspal delamination and attrition in molars, as well as chalky, fracture-prone incisors with irregular wear and disrupted dentin–enamel boundaries (Fig. 4a). Micro-CT confirmed significantly reduced enamel thickness (Fig. 4b, c), and scanning electron microscopy revealed disorganized hydroxyapatite crystallites with porous rod architecture and loss of the normal interwoven pattern (Fig. 4d). These findings demonstrate that *Slc4a9* deficiency impairs enamel structure and mineralization, extending its pathological impact beyond the salivary gland.

Beyond the Sjögren’s syndrome-like phenotype, *Slc4a9*^*−*^^*/−*^ mice exhibit enamel hypomineralization — a pathology previously attributed to the secondary effects of reduced salivary flow in patients with Sjögren’s syndrome^14^. Given the established primary roles of SLC4A family transporters (*Slc4a2*^*−*^^*/−*^ and *Slc4a4*^*−*^^*/−*^ mice exhibit intrinsic enamel defects) in enamel matrix secretion and mineralization, we aim to elucidate the primary function of *Slc4a9* in enamel development through analysis of tooth morphology in mice at distinct developmental stages. Using immunohistochemistry, we detected robust SLC4A9 expression in murine ameloblasts (Supplementary Fig. 4a). Strikingly, postnatal day 1 (P1) *Slc4a9*^*−*^^*/−*^ molars lacked discernible enamel matrices, with ameloblasts arrested in a pre-secretory state devoid of Tomes’ processes (Fig. 4e). In contrast, WT P1 molars exhibited active secretory-phase ameloblasts that produced organized enamel layers. Developmental delays were further evident in *Slc4a9*^*−*^^*/−*^ incisors, which showed a reduced crown size and hypoplastic enamel (Fig. 4e). In addition, embryonic staging results (E16.5 and E18.5) further revealed slightly delayed enamel development (Supplementary Fig. 4b), indicating early functional impairment. These data establish SLC4A9 as indispensable for ameloblast differentiation and matrix secretion, thereby uncoupling primary enamel defects from secondary salivary dysfunction in the autoimmune context.

### *Slc4a9* deficiency disturbs ameloblast development and enamel mineralization

**Fig. 5:**
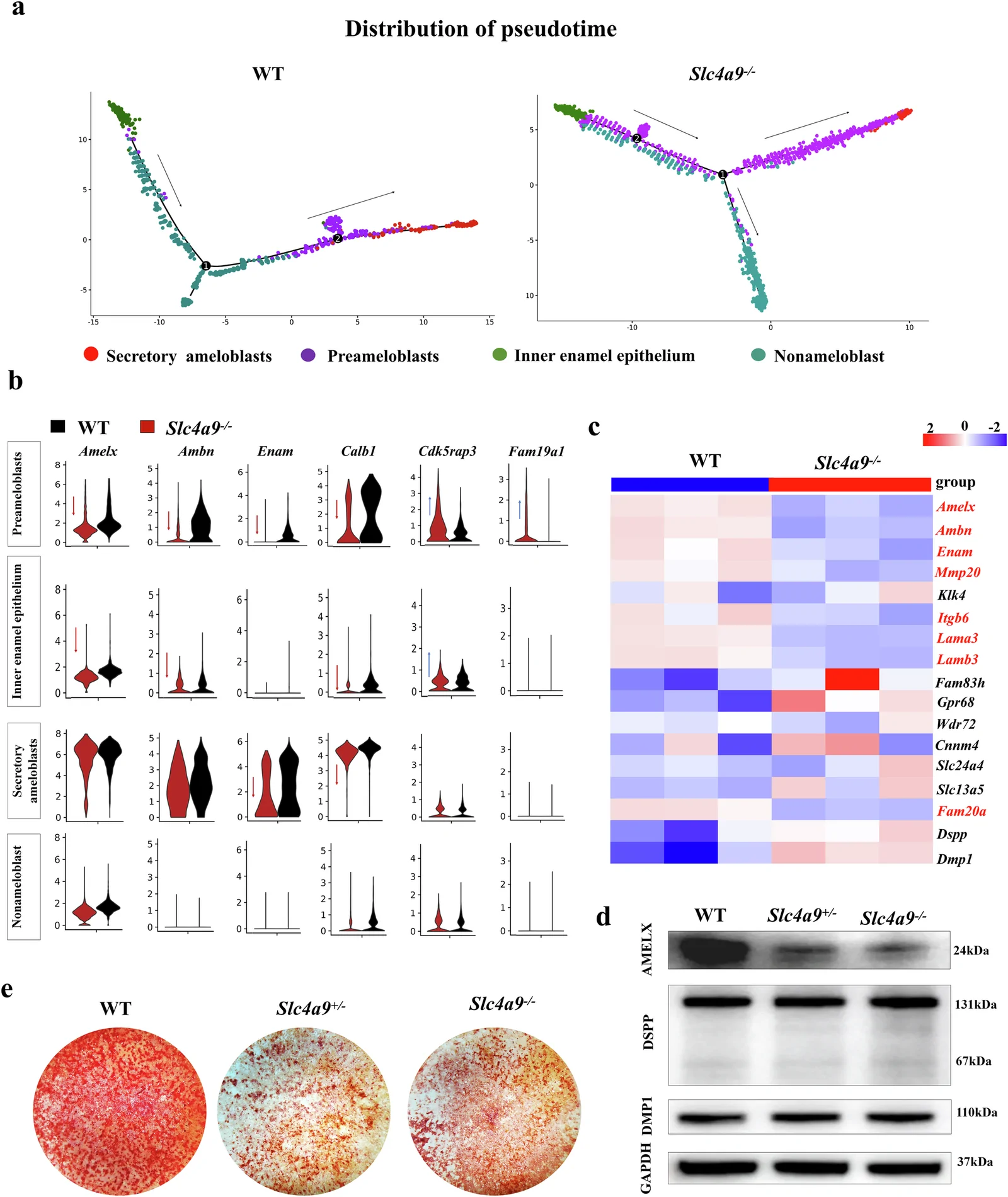
Delayed ameloblast development and reduced tooth calcification in *Slc4a9*^*−*^^*/−*^ teeth. **a** Altered ameloblast lineage progression. Single-cell trajectory analysis of wild type (WT) and *Slc4a9*^*−*^^*/−*^ ameloblasts. Pseudotime ordering revealed delayed differentiation in *Slc4a9*^*−*^^*/−*^ ameloblasts, with an accumulation of pre-ameloblasts and a reduction of secretory ameloblasts. Arrow indicates developmental trajectory. **b**, Downregulation of enamel-related markers. Violin plots show single-cell RNA sequencing (ScRNA-seq) expression levels of significantly altered genes in WT versus *Slc4a9*^*−*^^*/−*^ ameloblasts. Adjacent arrows indicate downregulation (red) or upregulation (blue). Enamel-related genes such as *Amelx*, *Ambn*, *Enam* and *Calb1* are downregulated in the *Slc4a9*^*−*^^*/−*^ group. **c** Dysregulated enamel gene expression. Heatmap of bulk RNA-seq analysis of postnatal day 1 (P1) tooth germs. Enamel development-related genes (e.g. *Amelx, Enam, Ambn, Mmp20*) are downregulated in *Slc4a9*^*−*^^*/−*^ compared with WT. **d** Specific loss of enamel matrix proteins. Immunoblot analysis of P1 molar lysates. AMELX expression is abolished in *Slc4a9*^*−*^^*/−*^ mice, whereas dentin markers (DSPP, DMP1) remain unchanged. β-actin serves as a loading control. **e** Impaired mineralization capacity. Alizarin red staining of primary cultured tooth germ cells after 21-day osteogenic induction. *Slc4a9*^*−*^^*/−*^ cultures exhibit reduced mineralized nodule formation compared with WT.

To define the cell-autonomous role of *Slc4a9* in amelogenesis, we performed single-cell RNA sequencing on P1 maxillary first molars (Supplementary Fig. 5a–d). Notably, ameloblasts, which differentiate from the inner enamel epithelium, undergo three developmental stages: pre-ameloblasts, secretory-stage ameloblasts and maturation-stage ameloblasts^30^. Pseudotime trajectory analysis reconstructed the trajectory of ameloblast fate in enamel development. Strikingly, *Slc4a9*^*−*^^*/−*^ mice exhibited a differentiation blockade, with significant accumulation of pre-ameloblasts and reduced secretory-stage ameloblasts (Fig. 5a).

The genes *AMELX* (amelogenin), *AMBN* (ameloblastin) and *ENAM* (enamelin) encode core enamel matrix proteins essential for dental-enamel biomineralization^31,32^. Mutations in these genes are a primary cause of amelogenesis imperfecta, a group of genetic disorders characterized by enamel hypoplasia, hypomineralization and structural fragility. The expression of these genes exhibits strict spatiotemporal specificity, primarily distributed in pre-ameloblasts and secretory-stage ameloblasts, regulating the formation, mineralization and structural stability of enamel^33^. Marker analysis revealed suppressed expression of enamel matrix genes (*Amelx, Ambn, Enam*) in *Slc4a9*^*−*^^*/−*^ secretory-stage ameloblasts and pre-ameloblasts, alongside aberrant persistence of molecules involved in cell-cycle regulation (*Cdk5rap3, Fam19a1*) in pre-ameloblasts (Fig. 5b), confirming stalled lineage progression consistent with our histological results.

Transcriptome sequencing and western blot analyses revealed significant downregulation of enamel-specific genes in *Slc4a9*^*−*^^*/−*^ mice, including *Amelx*, *Enam*, *Ambn*, *Mmp20* and *Klk4*, whereas dentin-related genes *Dspp* and *Dmp1* exhibited no significant expression differences, indicating selective impairment of enamel biomineralization pathways (Fig. 5c, d). Enamel matrix proteins are critical for hydroxyapatite nucleation and prismatic enamel organization. To confirm the effect of reduced enamel matrix protein content on enamel hardness, we primary cultured tooth germ cells in calcification-inducing medium. Mineralized nodule formation was inhibited in *Slc4a9*^*−*^^*/−*^ mouse cells compared with WT controls (Fig. 5e). This suggests that loss of *Slc4a9* not only delays enamel development but also inhibits tooth calcification. In conclusion, the enamel defect observed in *Slc4a9*^*−*^^*/−*^ mice is not secondary to reduced salivation but the primary defects due to *Slc4a9* deficiency.

### Loss of *Slc4a9* disturbs ion transport and pH regulation in ameloblasts and salivary cells

**Fig. 6:**
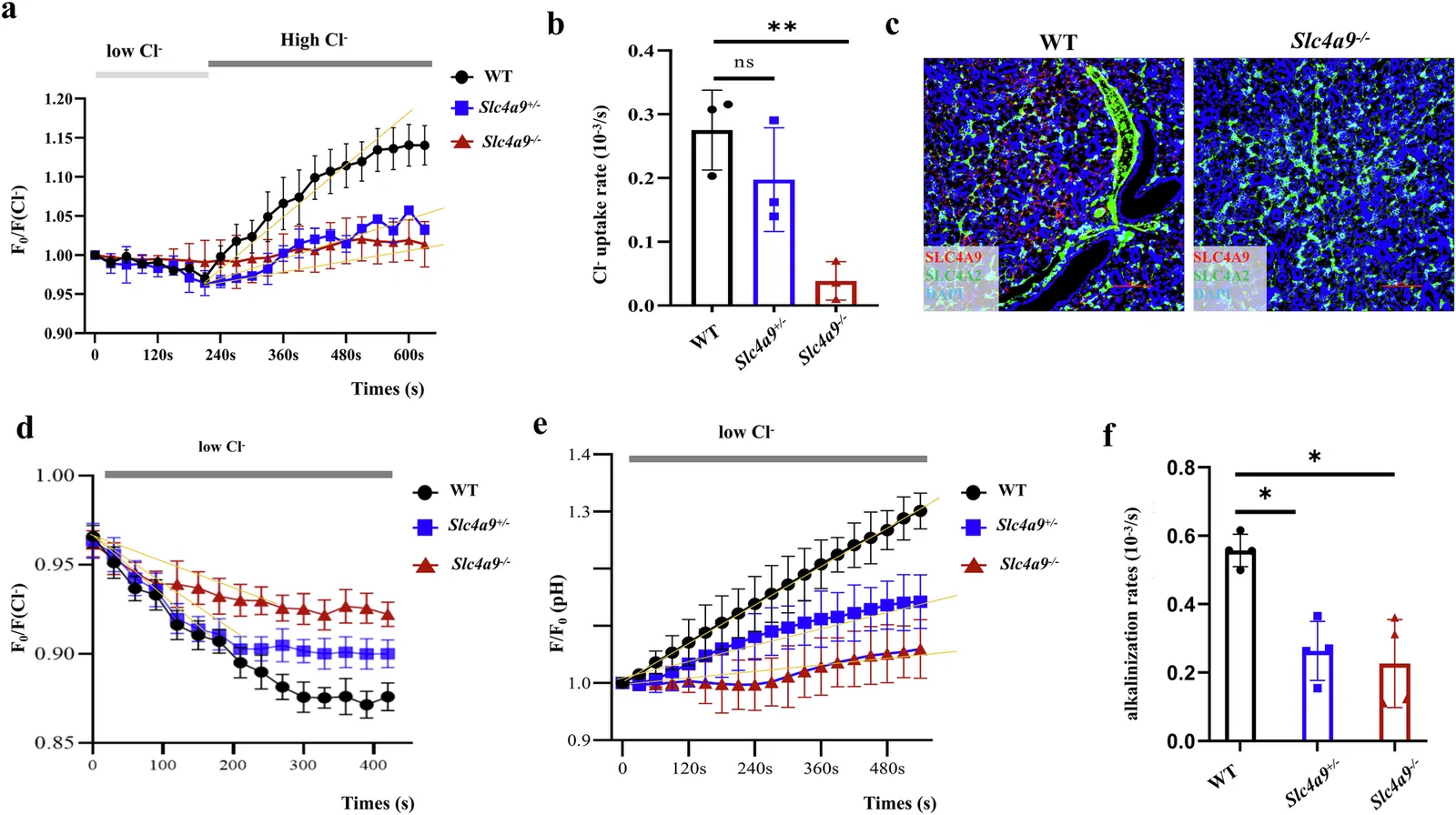
Changes in Cl transport and pH homeostasis in *Slc4a9*^*−*^^*/−*^ mice. **a** **b** Cl^−^ uptake experiments were performed in primary cultured salivary-gland cells from wild type (WT) and *Slc4a9* knockout mice. **a** After intracellular Cl^−^ was depleted in a low Cl^−^ bath solution, Cl^−^ uptake was initiated by the addition of high Cl^−^. **b** Summary of the initial Cl^−^ uptake rates calculated from experiments like those shown in **a** from the regions indicated with the red lines. The Cl^−^ uptake rate was significantly reduced in the *Slc4a9*^*−*^^*/−*^ group. Data are represented as individual points for each mouse. Statistical significance was determined by *t* test: **P < 0.01. **c** Immunofluorescence staining of SLC4A9 and SLC4A2 in primary cultured submandibular gland cells. SLC4A9 immunoreactivity (red) was detected in both acinar and ductal compartments; however, its signal intensity was notably weaker in the cytoplasm of *Slc4a9*^*−*^^*/−*^ cells compared with WT controls. In contrast, SLC4A2 expression (green) showed no significant difference between *Slc4a9*^*−*^^*/−*^ and WT mice. **d** Changes in Cl^−^ transport over time in primary cultured tooth germ cells. The yellow slash indicates the Cl^−^ transport rate (transition from high-chlorine solution to low-chlorine solution to observe the depletion of Cl^−^). The Cl^−^ uptake rate was also reduced in the *Slc4a9*^*−*^^*/−*^ group in tooth germ cells. **e** Cl^−^/HCO_3_^−^ exchange activity was initiated by switching from high-Cl^−^ to low-Cl^−^ bathing solutions (solution A to B), leading to a rapid intracellular alkalinization. Transport activity decreased by approximately 45% in the *Slc4a9*^*+*^^*/−*^ and ~55% in the *Slc4a9*^*−*^^*/−*^ group. **f** Summary of the initial alkalinization rates of experiments shown in **e**. Data are represented as individual points for each mouse. Statistical significance was determined by *t* test: *P < 0.05, **P < 0.01.

SLC4A9, a key regulator of Cl^−^/HCO_3_^−^ exchange, maintains intracellular pH homeostasis through its anion transport activity. To evaluate net Cl^−^/HCO_3_^−^ exchange within the glandular microenvironment, we performed functional assays using primary cultures of mixed salivary-gland cells. Cells were first exposed to a low-chloride solution, followed by a high-chloride solution to assess Cl^−^ uptake. WT cells exhibited a time-dependent increase in Cl^−^ uptake under high-chloride conditions, whereas *Slc4a9*^*−*^^*/−*^ cells showed a complete loss of this response (Fig. 6a). Consistently, quantitative analysis demonstrated a significant reduction in maximal Cl^−^ uptake velocity in *Slc4a9*^*−*^^*/−*^ cells compared with WT controls (Fig. 6b), confirming impaired Cl^−^ transport in the absence of *Slc4a9*. Previous studies have shown that simultaneous loss of SLC4A9 and SLC4A2 is required to abolish Cl^−^/HCO_3_^−^ exchange activity, suggesting functional compensation between these transporters. To investigate whether SLC4A2 is upregulated in response to SLC4A9 deficiency, we examined the expression of SLC4A9 and SLC4A2. As expected, SLC4A9 expression was reduced in both acinar and ductal compartments, whereas SLC4A2 levels showed no significant differences at either the RNA or the protein level between *Slc4a9*^*−*^^*/−*^ and WT mice (Fig. 6c, Supplementary Fig. 6). These results indicate that the ionic defects observed in *Slc4a9*^*−*^^*/−*^ mice are not attributable to increased SLC4A2 expression.

To define the role of *Slc4a9* in pH regulation within ameloblast-lineage cells, we examined its function using primary tooth germ cells. Cells were exposed to a low-chloride solution, which is expected to promote Cl^−^ efflux in exchange for HCO_3_^−^ influx via Cl^−^/HCO_3_^−^ exchangers, resulting in cytosolic alkalinization. Consistent with this model, Cl^−^ transport activity increased significantly in WT cells but remained low in *Slc4a9*^*−*^^*/−*^ cells (Fig. 6d). Moreover, upon Cl^−^ withdrawal, WT cells exhibited a rapid increase in intracellular pH, whereas *Slc4a9*^*−*^^*/−*^ cells showed a markedly attenuated alkalinization response (Fig. 6e, f). These findings identify *Slc4a9* as a key mediator of Cl^−^/HCO_3_^−^ exchange in ameloblasts. Its deficiency impairs the ability of cells to alkalinize the cytosol in response to Cl^−^ gradients, thereby disrupting pH regulation required for enamel mineralization. Together, these results indicate that, in addition to its role in salivary secretion, *Slc4a9* regulates enamel biomineralization through control of ion transport and intracellular pH homeostasis.

### Impaired Cl^−^ transport due to *Slc4a9* deficiency induces changes in RPS27 expression

**Fig. 7:**
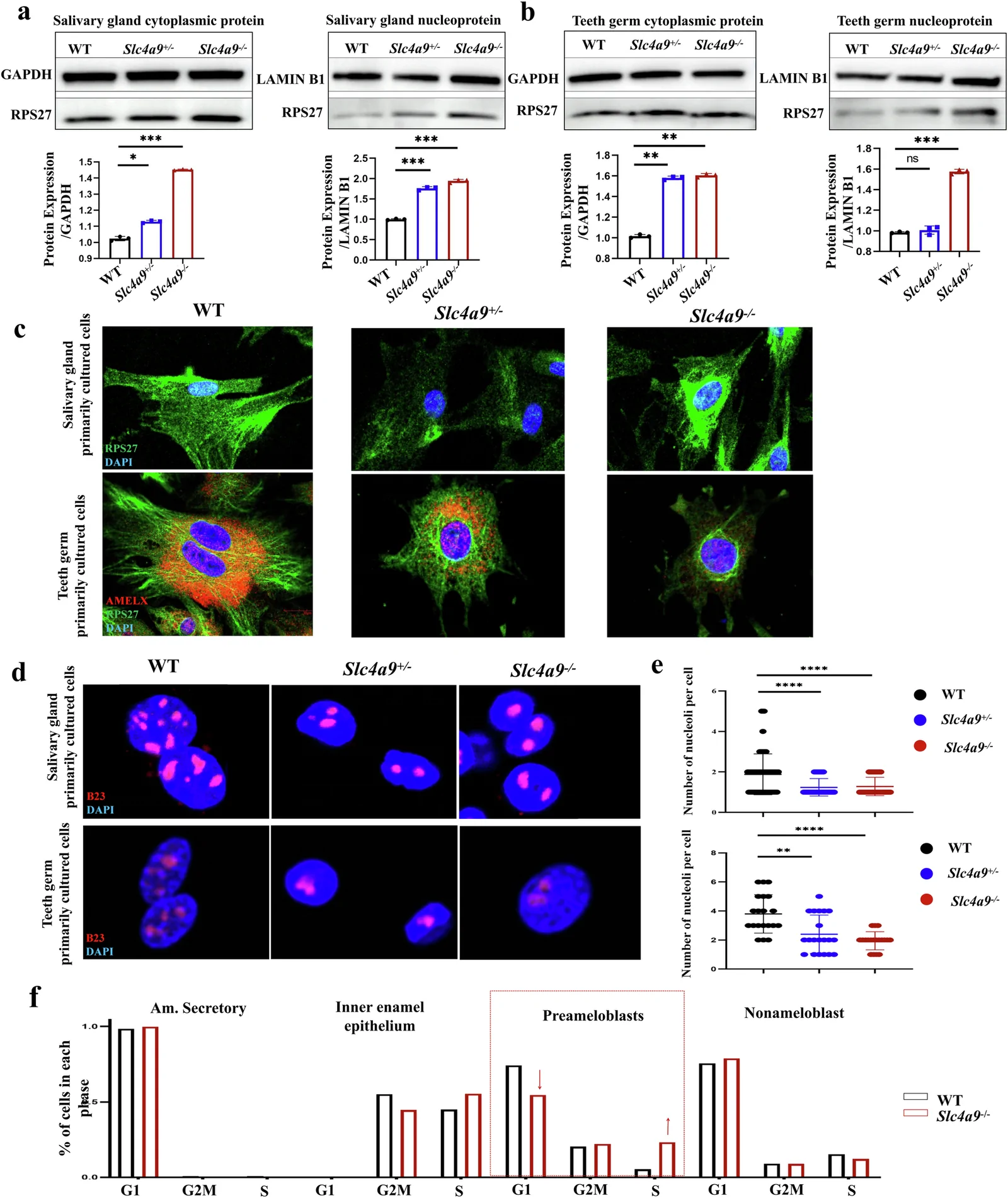
Changes in ribosomal gene *Rps27* in *Slc4a9*^*−*^^*/−*^ mice trigger nucleolar stress and cell-cycle arrest. **a** **b** RPS27 expression in submandibular gland (SMG) and tooth germ tissues. Western blot analysis of cytoplasmic and nuclear fractions, with quantification, showed increased RPS27 levels in both fractions from *Slc4a9*^*−*^^*/−*^ salivary glands (**a**) and tooth germs (**b**). Data are shown as mean ± SEM, *n* = 3; *P < *0.05*, **P < *0.01*, ***P < *0.001*; *t* test. **c**, Immunofluorescence staining of RPS27 in primary SMG and tooth germ cultures. AMELX was used to identify ameloblasts within heterogeneous tooth cells. RPS27 staining was increased in the cytoplasm of both SMG and tooth germ cells from *Slc4a9*^*−*^^*/−*^ mice compared with wild type (WT) controls. **d**, Immunofluorescence staining of nucleolar marker B23 and counterstained with DAPI in SMG and tooth germ cells. B23 positive signal: red; counterstain: DAPI/blue. **e** Quantification of B23-positive nucleoli per cell in SMG (*n* = 40 cells) and tooth germs (*n* = 20 cells). Data are represented as individual points for each nucleolus. Statistical significance was determined by *t* test: *P < 0.05, **P < 0.01, ***P < 0.01, ****P < 0.0001. **f** Cell-cycle profiles for each of the four clusters in tooth germ from single-cell RNA sequencing data. Red arrows highlight “up” or “down” trends in *Slc4a9*^*−*^^*/−*^ samples for the cell-cycle phase listed. The red box area indicates cell-cycle arrest in pre-ameloblasts.

Previous evidence suggests that changes in chloride levels may affect ribosomal genes such as *Rps27*^34^. RPS27, a component of the small ribosomal subunit, localizes to both the nucleus and cytoplasm. It interacts with DNA via its C4-type zinc finger domain and may facilitate translation initiation^35^. Transcriptome analysis in this study revealed high expression of *Rps27* in tooth germs and salivary-gland tissues, and numerous ribosome-related molecules, including *Rps27*, were significantly differentially expressed between WT and *Slc4a9*^*−*^^*/−*^ mice (Supplementary Fig. 7a, b). To examine changes at the protein level, we first performed immunohistochemical staining. In WT mice, RPS27 displayed a polarized distribution, with punctate apical cytoplasmic staining and minimal nuclear signal. In contrast, *Slc4a9*^*−*^^*/−*^ tissues showed disruption of this organized pattern, with diffuse RPS27 staining throughout the cell and increased nuclear signal (Supplementary Fig. 7c). To further quantify these alterations, we isolated nuclear and cytoplasmic proteins from salivary-gland and tooth germ tissues. Western blot analysis of the nuclear and cytoplasmic fractions revealed a significant increase in RPS27 expression in both compartments of *Slc4a9*^*−*^^*/−*^ mice (Fig. 7a, b). Consistent with these findings, immunofluorescence staining for RPS27 in primary cultured SMG cells and tooth germ cells further confirmed abnormal cytoplasmic accumulation of RPS27 in *Slc4a9*^*−*^^*/−*^ cells, with AMELX used as a specific marker to identify ameloblasts within the heterogeneous tooth cells (Fig. 7c). Taken together, these data demonstrate that SLC4A9 deficiency disrupts the polarized subcellular localization of RPS27.

To investigate the mechanism underlying the above observation, we considered that perturbations in rRNA synthesis, modification or ribosomal protein (r-protein) imbalance can disrupt ribosome biogenesis, leading to nucleolar stress and accumulation of free r-proteins^36,37^. Based on this, we speculate that SLC4A9 deficiency-induced RPS27 dysregulation impairs ribosome biogenesis, triggering nucleolar stress (ribosomal stress); the cytoplasmic accumulation of free RPS27 represents both a consequence and a biomarker of this stress response. To confirm this, we first employed immunofluorescent staining to determine the localization of B23 (nucleophosmin), a nucleolar protein involved in rRNA processing^38^. We found that the number of B23-labelled nucleoli was significantly reduced in both salivary-gland cells and tooth germ cells from *Slc4a9*^*−*^^*/−*^ mice, indicating impaired ribosome biogenesis and induced nucleolar stress (Fig. 7d, e). In summary, this section confirms that changes in RPS27 can trigger ribosomal stress in salivary-gland and tooth germ cells.

There is evidence that the disruption of ribosome biogenesis and/or the nucleolar structure activates p53-dependent or -independent signalling pathways, leading to cell-cycle arrest, apoptosis, differentiation and senescence^39^. We next analysed the distribution of four cell clusters in different cell cycles through single-cell sequencing cycles of tooth germs. Cell-cycle analysis shows an accumulation of cells in the S phase and fewer cells in G1 phase in *Slc4a9*^*−*^^*/−*^ mice, especially in pre-ameloblasts, indicating a cell-cycle arrest in S (Fig. 7f). Inhibition of apoptosis in ameloblasts in *Slc4a9*^*−*^^*/−*^ mice was observed by TUNEL staining (Supplementary Fig. 7d).

### Nutlin-3a restores salivary-gland mucin secretion in *Slc4a9*^*−*^^*/−*^ mice by rescuing dysfunction of the RPS27–MDM2–p53 axis

**Fig. 8:**
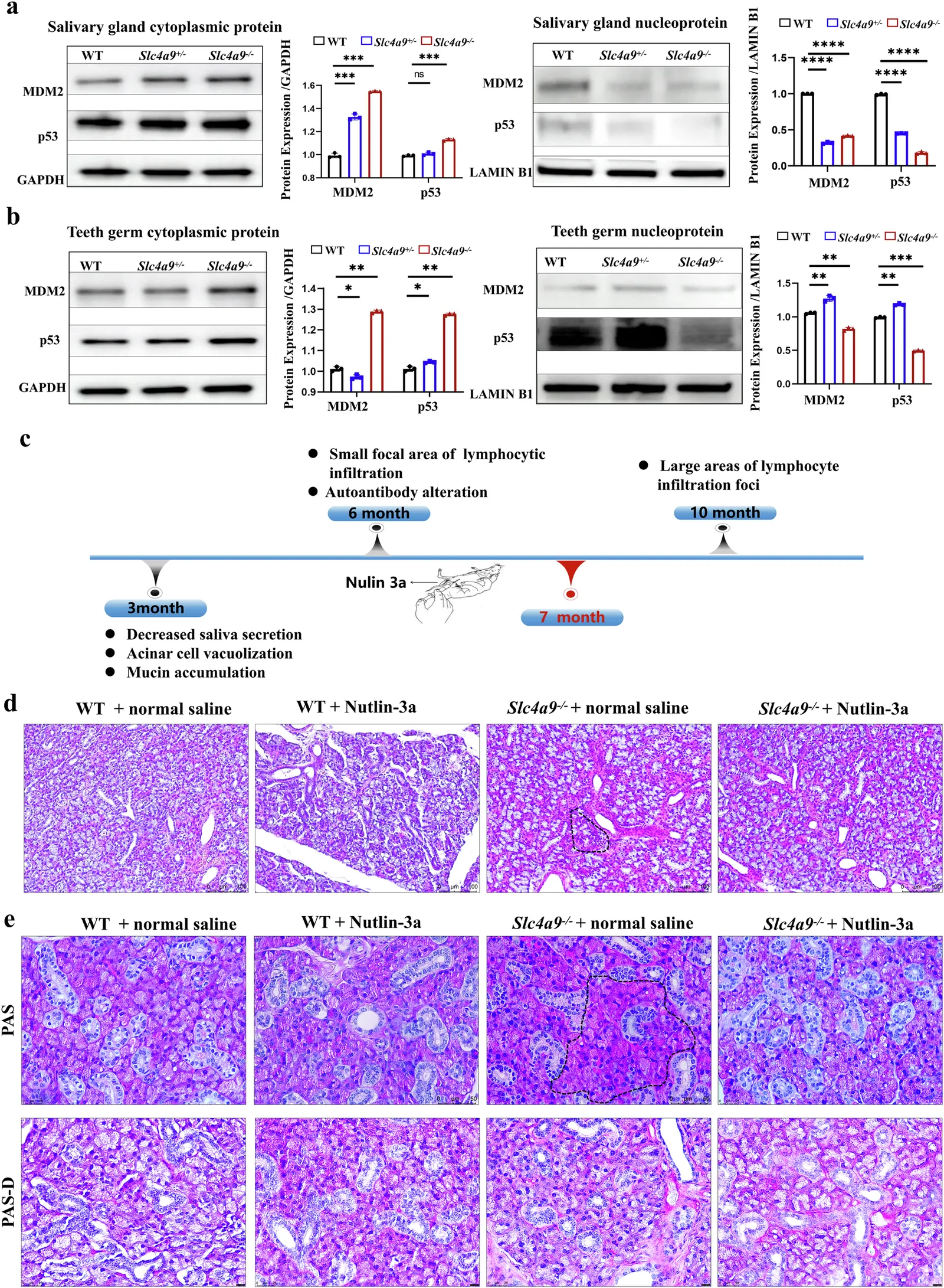
Nutlin-3a ameliorates salivary gland inflammatory phenotype in *Slc4a9*^*−*^^*/−*^ mice by rescuing dysfunction of the RPS27–MDM2–p53 axis. **a** **b** Western blot analysis of p53 and MDM2 expression in cytoplasmic and nuclear fractions of submandibular gland (SMG) (**a**) and tooth germ tissues (**b**) from wild type (WT) and *Slc4a9*^*−*^^*/−*^ mice. In *Slc4a9*^*−*^^*/−*^ tissues, p53 and MDM2 levels were significantly increased in the cytoplasm but decreased in the nucleus compared with WT levels. Mean values ± standard error of the mean are shown, *n* =  3 (*P < 0.05, **P < 0.01, ***P < 0.001, ****P < 0.0001, *t* test). **c** The timeline of Sjögren’s syndrome phenotype manifestation in *Slc4a9*^*−*^^*/−*^ mice. *Slc4a9*^*−*^^*/−*^ and WT mice in 6 months were treated with Nutlin-3a (20 mg/kg, every 48 h for 1 month) or vehicle-treated controls. Observe the phenotype of the submandibular gland in 7 months. **d** H&E-stained sections of SMGs from 7-month-old mice. Diffuse mononuclear-cell infiltrates (demarcated by dashed outlines) are evident in saline-treated *Slc4a9*^*−*^^*/−*^ mice. Nutlin-3a treatment reduced inflammatory infiltration. WT controls showed no pathological changes. **e** Periodic acid-Schiff (PAS) and diastase-digested PAS (PAS-D) staining of SMG sections from 7-month-old mice. Magenta-red staining highlights PAS-reactive material in acinar cells. Compared with controls, saline-treated *Slc4a9*^*−*^^*/−*^ mice exhibited excessive accumulation of PAS-reactive material (outlined by dotted lines), which was restored to near-control levels by Nutlin-3a treatment. Notably, diastase pretreatment markedly attenuated this signal specifically within the acini of saline-treated *Slc4a9*^*−*^^*/−*^ mice (PAS-D panel), consistent with the accumulation of digestible glycogen.

In response to ribosomal stress, free ribosomes bind to MDM2 and block MDM2-mediated p53 ubiquitination and degradation, inducing p53-dependent cell-cycle arrest^40^. Here, we investigated whether free RPS27, in response to nucleolar stress, similarly binds to MDM2 and inhibits p53 ubiquitination and degradation. Analysis of RPS27, p53 and MDM2 expression in nuclear and cytoplasmic fractions revealed reduced nuclear and increased cytoplasmic p53 levels in both salivary glands and tooth germs of *Slc4a9*^*−*^^*/−*^ mice (Fig. 8a, b). These data suggest that free RPS27 in the cytoplasm may competitively bind to MDM2 with p53, thereby inhibiting the ubiquitination and further degradation of p53.

Sjögren’s syndrome is an autoimmune disease that progresses over time. The timing of treatment selection is critical for effectively managing the disease. In ≤ 1-month-old *Slc4a9*^*−*^^*/−*^ mice, basolateral Cl^−^/HCO_3_^−^ exchange activity was reduced by 25% compared with WT, reflecting early epithelial ion homeostasis disruption without histopathological hallmarks of Sjögren’s syndrome. At 3 months, *Slc4a9*^*−*^^*/−*^ mice demonstrated secretory dysfunction, with a 25% reduction in saliva flow compared with WT mice, alongside acinar-cell shrinkage and vacuolation (Fig. 8c). Based on previous reports that the mouse model of Sjögren’s syndrome first manifests between 4 and 6 months of age^21^, combined with the observation of lymphocyte infiltration at 6 months in this study (Result 3), we established 4 months and 6 months as a critical node for intercepting Sjögren’s syndrome progression. Salivary-gland phenotype recovery was then assessed at the subsequent timepoints of 5 months and 7 months (Fig. 8c and Supplementary Fig. 8a).

Based on nucleolar stress and cell-cycle dysregulation induced by abnormal RPS27 accumulation, we performed rescue experiments with the MDM2 antagonist Nutlin-3a to validate its pivotal role in restoring p53 homeostasis and mitigating pathological phenotypes. After administration of Nutlin-3a (20 mg/kg, intraperitoneal injection every 48 h for 1 month) to *Slc4a9*^*−*^^*/−*^ mice, pathological damage of the salivary gland appeared to be alleviated. Histological analysis by H&E staining revealed mild lymphocyte infiltration in the SMGs of saline-treated *Slc4a9*^*−*^^*/−*^ mice, whereas such inflammatory infiltration was not clearly observed in the Nutlin-3a-treated group (Fig. 8d). Notably, conventional PAS staining revealed marked accumulation of PAS-reactive cytoplasmic material in the salivary glands of saline-treated *Slc4a9*^*−*^^*/−*^ mice; this accumulation was substantially reduced following Nutlin-3a treatment (Fig. 8e and Supplementary Fig. 8b). To determine the nature of this accumulation, we performed diastase digestion controls (PAS-D). Consistent with glycogen deposition, the intense cytoplasmic positivity in saline-treated *Slc4a9*^*−*^^*/−*^ mice was markedly diminished after diastase pretreatment (Fig. 8e), indicating the accumulated material as predominantly digestible glycogen. ELISA analysis of serum autoantibodies in the four experimental groups showed that although anti-SSB antibody levels did not change significantly in the Nutlin-3a-treated group, anti-SSA antibody levels were reduced, particularly in female mice tested at 7 months of age (Supplementary Fig. 8c). Together, these results suggest that Nutlin-3a may have potential therapeutic value in alleviating salivary-gland injury mediated by abnormalities in the *Slc4a9–Rps27–p53* pathway.

### Pathogenic *SLC4A9* variants identified in patients with amelogenesis imperfecta

Currently, at least 70 genes are reported to be associated with amelogenesis imperfecta, which involves genes encoding enamel-matrix proteins, enamel-matrix proteases, cell-adhesion molecules, transporters or pH sensors and developmental regulatory molecules, etc.^33,41^. Given that *Slc4a9* knockout mice exhibit enamel defects, we investigated whether *SLC4A9* represents a novel causative gene for amelogenesis imperfecta. Three patients diagnosed with amelogenesis imperfecta were enrolled in this study. Patient 1 and Patient 2 are siblings, whereas Patient 3 is a sporadic case with no available family history (Supplementary Fig. 9a).

**Fig. 9:**
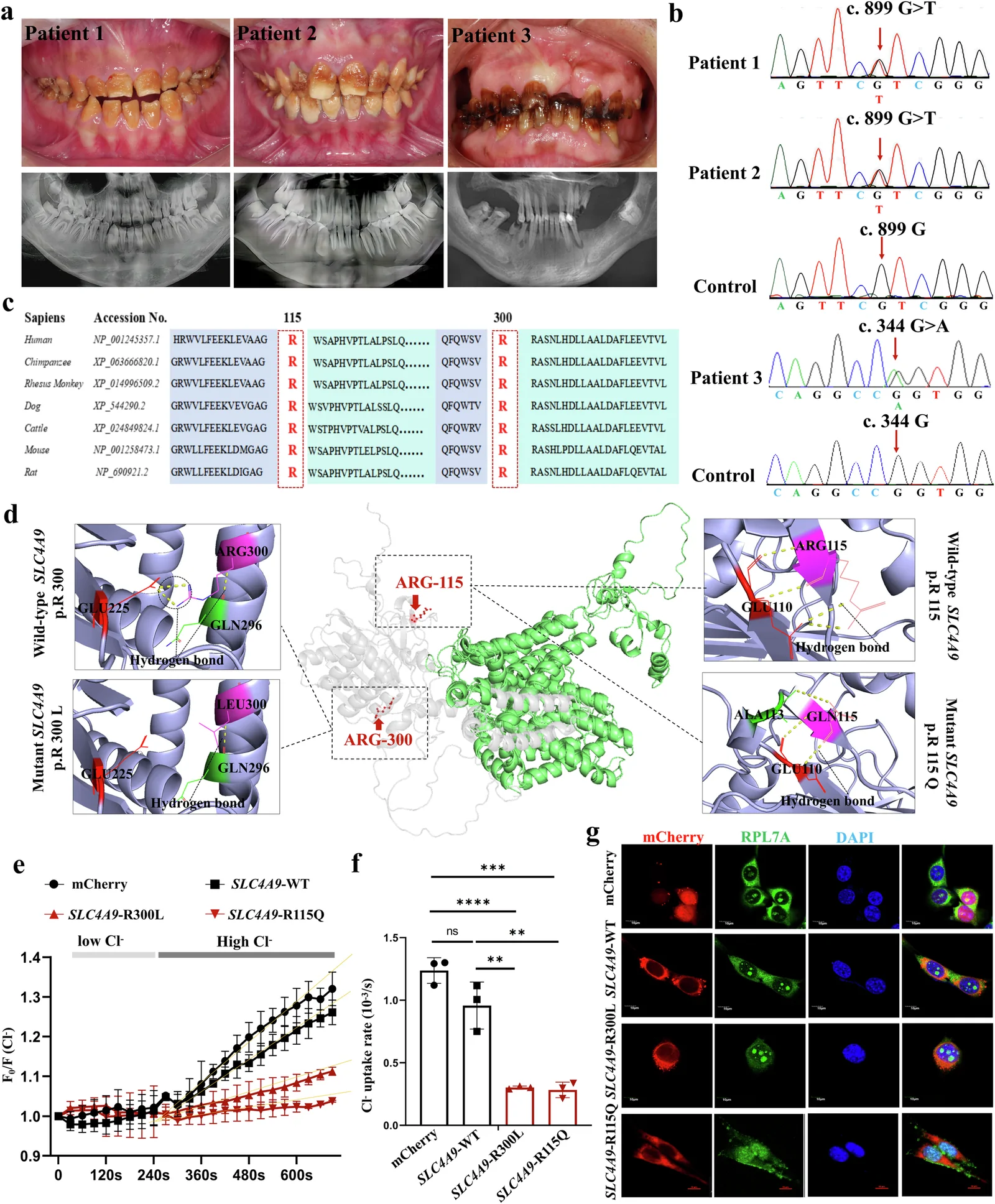
*SLC4A9* variants identified in patients with amelogenesis imperfecta. **a** Clinical and radiographic features. Intraoral photographs and panoramic radiographs of three patients with amelogenesis imperfecta (Cases 1–3) show generalized enamel hypoplasia and thinning. Cases 1 and 2 are siblings. Case 3 was a sporadic case without family information. **b** Genetic validation. Validation of *SLC4A9* missense variants. Sanger sequencing chromatograms confirm two pathogenic missense variants in *SLC4A9* (NM_001258428.2): c.899 G > T (p. Arg300Leu) in Cases 1 and 2 and c.344 G > A (p. Arg115Gln) in Case 3. **c** Evolutionary conservation. Multiple sequence alignment of vertebrate orthologues reveals that arginine residues at positions 115 and 300 (red boxes) are 100% conserved across species (from human to rat). **d** Structural perturbations of *SLC4A9* variants. Predicted 3D structures show how the p.R300L and p.R115Q mutations disrupt key hydrogen bonds (yellow dashed lines). In the central cartoon, the green region represents the transmembrane domain of the protein, whereas both mutated arginine residues are located in non-transmembrane regions. Magnified views of each site compare wild type and mutant structures, highlighting the loss of hydrogen-bond interactions upon mutation. **e** Reduced chloride uptake in ameloblasts. LS8 ameloblast cells were transfected to overexpress *SLC4A9-WT, SLC4A9-R300L* or *SLC4A9-R115Q*. Intracellular Cl^−^ was first depleted in a low-Cl^−^ bath solution, and Cl^−^ uptake was initiated by adding a high-Cl^−^ solution. **f**, The summary of initial Cl^−^ uptake rates, calculated from the time interval indicated by the yellow line in the representative traces of **e** shows that both the *SLC4A9*-R300L and *SLC4A9*-R115Q groups have lower Cl^−^ uptake rates than the mCherry control and *SLC4A9*-WT groups. **g** Altered subcellular localization. Immunofluorescence staining was performed to assess the impact of SLC4A9 mutation on the localization of the ribosomal marker RPL7A. Confocal immunofluorescence imaging of LS8 cells revealed increased nuclear localization of RPL7A in the overexpression of *SLC4A9*-R115Q and *SLC4A9*-R300L.

The proband and her brother in pedigree 1 exhibited severe enamel hypocalcification, characterized by soft, friable enamel with widespread detachment that exposed the underlying dentin and resulted in brown–black discoloration. Significant crown-height reduction and an irregular surface texture were consistent with advanced enamel disintegration. Patient 3 displayed analogous enamel defects but with deeper pigmentation, suggesting mineralization failure (Fig. 9a). Radiographic analysis revealed a diminished radiodensity contrast between enamel and dentin in all patients. Unerupted teeth demonstrated ragged enamel margins and reduced radiopacity, aligning with histological features of enamel hypoplasia (Fig. 9a).

Genomic DNA from the peripheral blood of the three patients and their family members was used for whole-exome sequencing analysis. Two missense variants c.899 G > T (p.R300L) and c.344 G > A (p.R115Q) in *SLC4A9* (NM_001258428.2) were detected in three cases (Fig. 9b). All mutations segregated with the disease (Supplementary Fig. 9a). The prevalence of c.899 G > T (p.R300L) is 2/1613884 and c.344 G > A (p.R115Q) is 269/1613092 in the general population (gnomAD). Two variants were predicted to be damaging by the PolyPhen2 and Sift algorithms.

The arginine residues R300 and R115 in *SLC4A9* are evolutionarily conserved across mammalian species (Fig. 9c), reflecting their critical roles in maintaining protein stability and ion transport activity. Structural modelling indicates that these residues are located in the cytoplasmic region near the SLC4A9 N-terminus, rather than within the transmembrane segments. In the wild type structure, Arg300 forms three β-sheet-like hydrogen bonds with Glu225, whereas Arg115 engages Glu110 via four similar hydrogen bonds, interactions that contribute to the structural stability of SLC4A9 (Fig. 9d). In the R300L and R115Q mutants, substitution of the charged guanidino group of arginine with the hydrophobic side chain of leucine or the polar but uncharged side chain of glutamine abolishes charge complementarity and substantially reduces hydrogen-bonding potential (Fig. 9d). The instability of charge and the reduction of hydrogen bonds both lead to the destabilization of protein structure^42,43^. Hence, we speculate that the R300L and R115Q variants may act by reducing the β-sheet structure, ultimately impairing protein stability, which directly impairs the Cl^−^/HCO_3_^−^ exchange activity of SLC4A9.

Recent evidence indicates that residues T756 and T448 of human SLC4A9 are located within transmembrane regions involved in regulating ion transport. The double mutant T756A–T448I completely loses transport capability in the presence of extracellular Na^+^, yet retains anion transport activity when K^+^ serves as the predominant extracellular cation^44^, suggesting that mutations at these key sites critically influence the ion transport function of SLC4A9. To clarify the biological relevance of mutations screened in patients with amelogenesis imperfecta, we overexpressed *SLC4A9*-WT, *SLC4A9*-R300L and *SLC4A9*-R115Q in the LS8 ameloblast cell line. Immunoblotting confirmed comparable protein-expression levels of all three constructs (Supplementary Fig. 9b, c). Assessment of chloride transport via uptake assays revealed that cells overexpressing *SLC4A9*-WT exhibited a response similar to that of mCherry-expressing controls, probably reflecting the high baseline of endogenous SLC4A9-dependent transport activity in LS8 cells. In contrast, cells expressing the R300L or R115Q mutants showed a marked reduction in chloride uptake to levels significantly below those of the mCherry control (Fig. 9e, f). Given the equivalent protein expression, these data suggest that the R300L and R115Q variants impair chloride transport, potentially by interfering with endogenous SLC4A9 activity, rather than merely representing loss-of-function alleles. Mechanistically, immunofluorescence staining revealed increased nuclear localization of the ribosomal marker RPL7A in LS8 cells overexpressing *SLC4A9*-R115Q or *SLC4A9*-R300L relative to *SLC4A9*-WT-expressing cells (Fig. 9g). Consistent with this, western blot analysis demonstrated upregulation of RPS27 and activation of the p53–MDM2 axis in *SLC4A9*-mutant LS8 cells (Supplementary Fig. 9d, e). These in vitro findings corroborate our prior in vivo observations, further validating the functional impact of these mutations.

## Discussion

This study establishes a close relationship between SLC4A9 and Sjögren’s syndrome by demonstrating reduced SLC4A9 levels and altered *SLC4A9* variants in cases of Sjögren’s syndrome. Furthermore, we innovatively generated Sjögren’s syndrome-like phenotypes in *Slc4a9*^*−*^^*/−*^ mice, providing new insights into the role of Cl^−^/HCO_3_^−^ exchangers in salivary function. We also identified *SLC4A9* as a causative gene for amelogenesis imperfecta and demonstrated that *SLC4A9* deficiency directly leads to enamel defects, independently of hyposalivation.

High intracellular Cl^−^ levels in salivary-gland acinar cells are essential for primary saliva secretion^8^. SLC4A9 mediates Na^+^-dependent Cl^−^/HCO_3_^−^ exchange, whereas NKCC1 (SLC12A2) facilitates electroneutral Cl^−^ influx^45^. Together, these transporters coordinate the Cl^−^ homeostasis required for salivary secretion. SLC4A2 and SLC4A9 are major Cl^−^/HCO_3_^−^ exchangers in the salivary gland^11,46^, whereas SLC26 family members contribute to apical ion exchange and pH regulation. Disruption of this coordinated network may underlie glandular dysfunction in Sjögren’s syndrome. Notably, we observed loss of SLC4A9 immunoreactivity in Sjögren’s syndrome-afflicted salivary glands, suggesting that impaired Cl^−^/HCO_3_^−^ exchange contributes to disease pathogenesis. Consistently, *Slc4a9*^*−*^^*/−*^ mice exhibited reduced saliva secretion, inflammatory infiltration and female-biased autoantibody production, recapitulating key features of Sjögren’s syndrome^47^. No compensatory changes in *Slc4a2* or *Slc4a4* were detected, highlighting the non-redundant role of SLC4A9.

This study further suggests that SLC4A9 deficiency not only disrupts ion homeostasis but may also impair ribosome biogenesis. RPS27, a component of the 40S ribosomal subunit, is involved in ribosome assembly and protein synthesis. Under normal conditions, ribosomal proteins are synthesized in the cytoplasm and transported to the nucleolus for assembly with rRNA into pre-ribosomal complexes^48^. In contrast, ribosomal stress caused by impaired biogenesis leads to the accumulation of unassembled ribosomal components in the cytoplasm^40,49^. Consistent with this, we observed altered expression and subcellular localization of RPS27 in *Slc4a9*-deficient tissues, suggesting disrupted ribosome assembly. These findings support a model in which SLC4A9 deficiency induces ribosomal stress, contributing to salivary-gland damage and enamel defects.

**Fig. 10:**
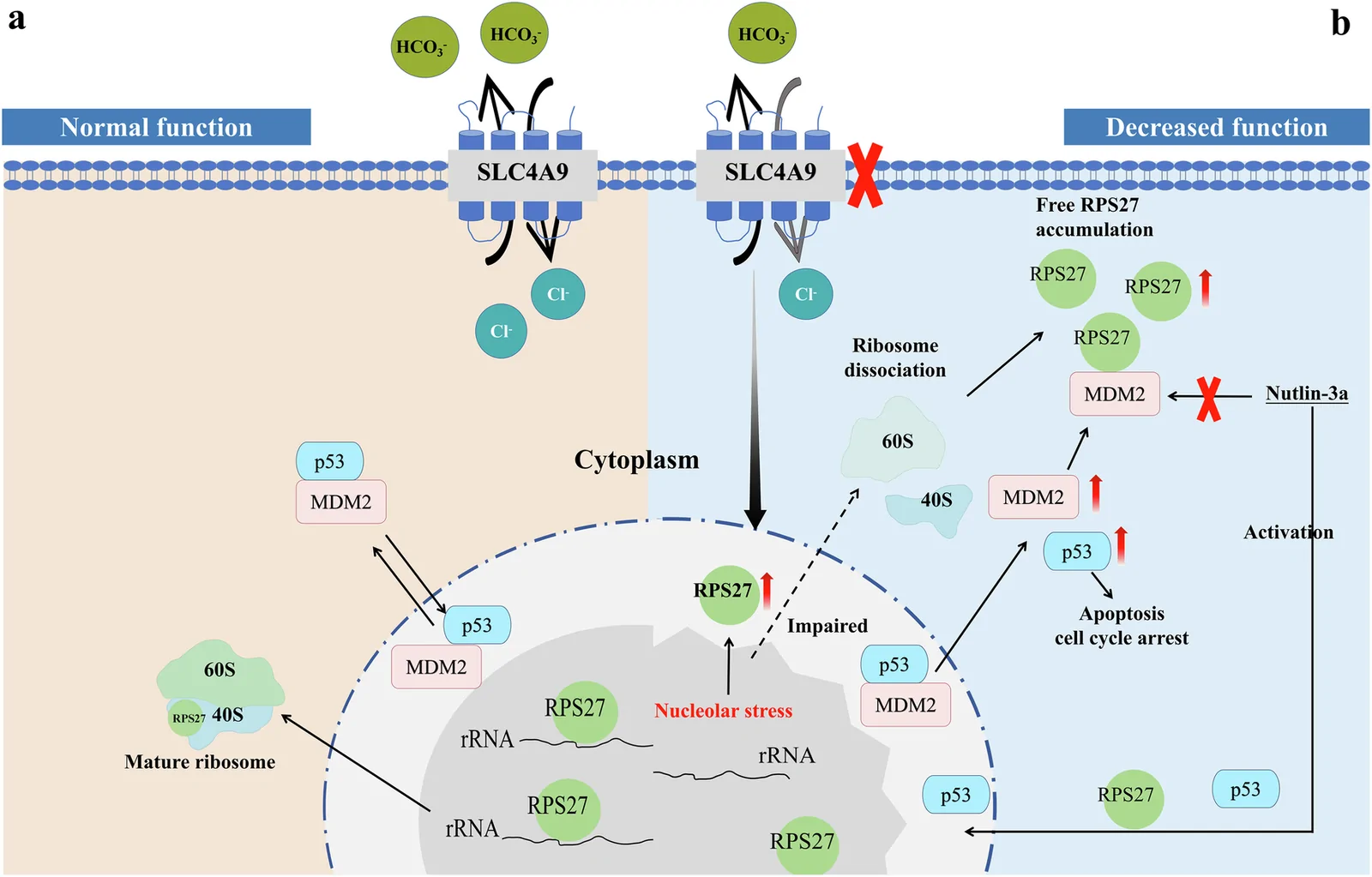
Schematic map showing SLC4A9-related ribosomal stress. **a** SLC4A9 mediates Na^+^-dependent Cl^−^/HCO_3_^−^ transport under normal conditions, which maintains ion and pH homeostasis inside and outside cells. RPS27 is involved in translation processes and ribosomal small subunit biogenesis. **b** SLC4A9 deficiency changes ion and pH homeostasis, leading to the accumulation of RPS27 in the cytoplasm, which further causes impaired ribosome biogenesis and induced nucleolar stress. In response to ribosomal stress, free ribosomes (RPS27) bind to MDM2 and block MDM2-mediated p53 ubiquitination and degradation, resulting in p53-dependent cell-cycle arrest and apoptosis. The selective MDM2 antagonist Nutlin-3a binds to MDM2 in the RPS27-binding pocket, disrupts MDM2–RPS27 interaction, and increases both nuclear and cytoplasmic p53 and RPS27 levels.

It is well established that perturbed ribosome biogenesis triggers the release of ribosomal proteins (RPs), including RPL5 and RPL11, from the nucleolus to the nucleus. There, they bind MDM2, inhibiting its ubiquitin ligase activity towards p53 and leading to p53 accumulation^40^. Our findings reveal a paradoxical p53 activation pattern in SLC4A9-deficient models: cytoplasmic accumulation of active p53 coupled with nuclear depletion. This spatial dichotomy challenges the canonical nucleolar stress–MDM2–p53 axis, suggesting a possible cytoplasm-centric regulatory mechanism. Previous reports indicate that MDM2 promotes RPS27 ubiquitination under conditions of overexpression but not under physiological conditions^49^. In this study, SLC4A9 deficiency induced RPS27 cytoplasmic enrichment that may create a permissive micro-environment for MDM2–RPS27 interactions. Therefore, we speculate that MDM2 may bind to RPS27 in the cytoplasm, promoting p53 activation (Fig. 10). Furthermore, treatment with Nutlin-3a rescued the impaired salivary-gland phenotype, including mucin expression and cellular vacuolization, in *Slc4a9*^*−*^^*/−*^ mice. This phenotypic rescue confirms the dual role of MDM2 as a suppressor of p53 in the nucleus and a stabilizer of p53 in the cytoplasm within ribosome-stressed microenvironments. The compartment-specific effects of Nutlin-3a, which preferentially inhibits nuclear MDM2–p53 interactions, underscore the therapeutic potential of spatially targeted MDM2 inhibitors.

Amelogenesis imperfecta refers to a genetically and clinically heterogeneous group of conditions characterized by inherited developmental enamel defects, including abnormalities in enamel colour, thickness and mineralization^41^. In this study, we report, to our knowledge for the first time, that *SLC4A9* is a novel causative gene for amelogenesis imperfecta. Building on prior evidence linking pH dysregulation to enamel defects in *Slc4a2*^*−*^^*/−*^ and *Slc4a4*^*−*^^*/−*^ mouse models^50^, we demonstrate that *SLC4A9* deficiency disrupts Cl^−^/HCO_3_^−^ exchange in ameloblasts, leading to impaired pH homeostasis and defective chloride uptake during enamel development. Crucially, this ionic imbalance activates a p53-dependent ribosomal stress pathway, resulting in cell-cycle arrest in ameloblasts. The conserved function of SLC4 transporters in ion homeostasis is further underscored by *SLC4A9* variants that disrupt β-sheet structures and compromise protein stability. In ameloblasts, loss of SLC4A9-mediated Cl^−^ uptake perturbs pH regulation and impairs the processing of enamel-matrix proteins. For *SLC4A9*-related amelogenesis imperfecta, targeted interventions such as local chloride supplementation or AAV-mediated gene delivery may represent promising therapeutic strategies for restoring pH gradients, although further studies are warranted to validate their efficacy.

*Slc4a9*^+/^^−^ mice exhibited mild enamel defects compared with homozygous knockouts, consistent with a gene dosage effect and haploinsufficiency. In contrast, we found that heterozygous *SLC4A9* variants in humans were associated with severe enamel phenotypes, suggesting a dominant-negative mechanism. This discrepancy between mouse and human phenotypes may reflect differences in mutation type or species-specific sensitivity to SLC4A9 dysfunction. Together, these findings indicate that SLC4A9 does not follow a simple recessive inheritance pattern but instead involves more complex genetic mechanisms. This has important implications for genetic screening, as pathogenic variants may manifest in heterozygous carriers, and highlights the need to consider both haploinsufficiency and dominant-negative effects when interpreting SLC4A9-associated disease.

One of our intriguing new findings concerns the relationship between Sjögren’s syndrome and enamel defects under SLC4A9 deficiency. In recent years, several studies have reported an association between enamel defects and autoimmune diseases^51–53^. For example, enamel abnormalities have been observed in patients with autoimmune polyendocrinopathy–candidiasis–ectodermal dystrophy and coeliac disease^51,54,55^. It has been proposed that autoantibodies targeting ameloblast-specific proteins may disrupt enamel formation in autoimmune conditions. Additionally, autoimmune amelogenesis imperfecta specifically refers to enamel hypoplasia associated with IgA-dependent autoimmune disease^56^. However, a direct link between Sjögren’s syndrome and enamel defects has not been clearly established. In this study, *Slc4a9*^*−*^^*/−*^ mice provided compelling evidence for the connections between enamel and salivary gland. The abnormal changes in *Slc4a9*^*−*^^*/−*^ enamel and salivary gland share a similar ribosomal pathway, which may be related to a common ectodermal origin^12^, but it seemed that these changes occurred independently. *Slc4a9* deficiency directly affected enamel development in mice, contrary to enamel defects in patients with primary Sjögren’s syndrome, which are often secondary to hyposalivation^13,15,57^. This independence was further supported by human data: three patients with amelogenesis imperfecta with *SLC4A9* mutations showed no significant reduction in salivary secretion, whereas cases of primary Sjögren’s syndrome with intronic deletions in SLC4A9 displayed normal enamel morphology. Therefore, we propose the following explanation that *SLC4A9* is a new causative gene of amelogenesis imperfecta in a monogenic way, and contributes to the pathogenesis of primary Sjögren’s syndrome through more complex mechanisms.

In recent years, advances in sequencing technology have provided increasing evidence that Sjögren’s syndrome is associated with genetic variations. Genetic studies in primary Sjögren’s syndrome have identified mutations in genes such as *HLA, IRF5, STAT4, GTF2I, IRAK1BP1, ITSN2* and *CCL11*, which are involved in both innate and adaptive immune responses^7,58,59^. Additionally, potential susceptibility loci in *SLC26A4* have been reported in patients with primary Sjögren’s syndrome-associated pulmonary arterial hypertension, suggesting that pathogenic genes may serve as genetic markers for early detection of the disease^60^. In this study, we identified deletion copy number variation of *SLC4A9* in patients with primary Sjögren’s syndrome, suggesting that *SLC4A9* may represent a potential genetic marker for Sjögren’s syndrome.

## Supplementary information

Supplementary Information
