# Supplementary material for: SLC4A9 deficiency is involved in Sjögren’s syndrome and enamel defects through RPS27-triggered ribosomal stress
Source: Exp Mol Med. 2026 Oct 9;58(10):3546–64. doi: 10.1038/s12276-026-01867-0 (PMC13737185; doi:10.1038/s12276-026-01867-0)
Supplement: Supplementary file 1 [file 12276_2026_1867_MOESM1_ESM.pdf]

SLC4A9 deficiency is involved in Sjögren's syndrome and enamel defects through RPS27 triggered ribosomal stress

Wenyan Ruan1[#], Jing Dong1[#], Yali Xu1[#], Yanli Zhang1, Yulong Zhu1, Songya Mao1, Yuan Liu2, Xiaohong Duan1*

1State Key Laboratory of Oral & Maxillofacial Reconstruction and Regeneration
National Clinical Research Center for Oral Diseases
Shaanxi Key Laboratory of Stomatology
Department of Oral Biology & Clinic of Oral Rare Diseases and Genetic Diseases, School of Stomatology the Fourth Military Medical University, Xi'an, China;
2 Department of Oral Pathology, School of Stomatology the Fourth Military Medical University, Xi'an, China

# Contribute equally in this work

Email address：Wenyan Ruan (2225746719@qq.com), Jing Dong (571074161@qq.com), Yali Xu (a2258879785@126.com), Yanli Zhang (yanlizhang@fmmu.edu.cn), Yulong Zhu (630242308@qq.com), Songya Mao (sy.mao@foxmail.com), Yuan Liu (914718019@qq.com), Xiaohong Duan (xhduan@fmmu.edu.cn).

***Corresponding author:** Prof. Xiaohong Duan, The Fourth Military Medical University, Xi'an, Shaanxi, China. Email: xhduan@fmmu.edu.cn. Telephone: 029-84776174

Supplementary Information

Supplementary Fig. 1 Analysis of CNV deletion region in human *SLC4A9*.
Supplementary Fig. 2 *Slc4a9* knockout alters glycogen metabolism in salivary glands.
Supplementary Fig. 3 Age-related alterations and glycoprotein network changes in mouse salivary glands.
Supplementary Fig. 4 *Slc4a9* deficiency affects enamel development in mice.
Supplementary Fig. 5 Single-cell sequencing of 1-day postnatal mouse maxillary first molars.
Supplementary Fig. 6 Heat map of SLC4 family members in teeth germ tissue and submandibular salivary glands.
Supplementary Fig. 7 Ribosomal stress and apoptotic priming in *Slc4a9*-deficient secretory epithelia.
Supplementary Fig. 8 Nutlin-3a restores salivary gland inflammatory phenotype in *Slc4a9*$^{-/-}$ mice by reversing RPS27-MDM2-p53 axis dysfunction.
Supplementary Fig. 9 Establishment of overexpression *SLC4A9* mutant cell lines.

Supplementary Table 1 Sjögren's Syndrome diagnostic score based on combined European-American consensus
Supplementary Table 2 Mass spectrometric data for selected 40 O-glycans obtained from mouse submandibular glands

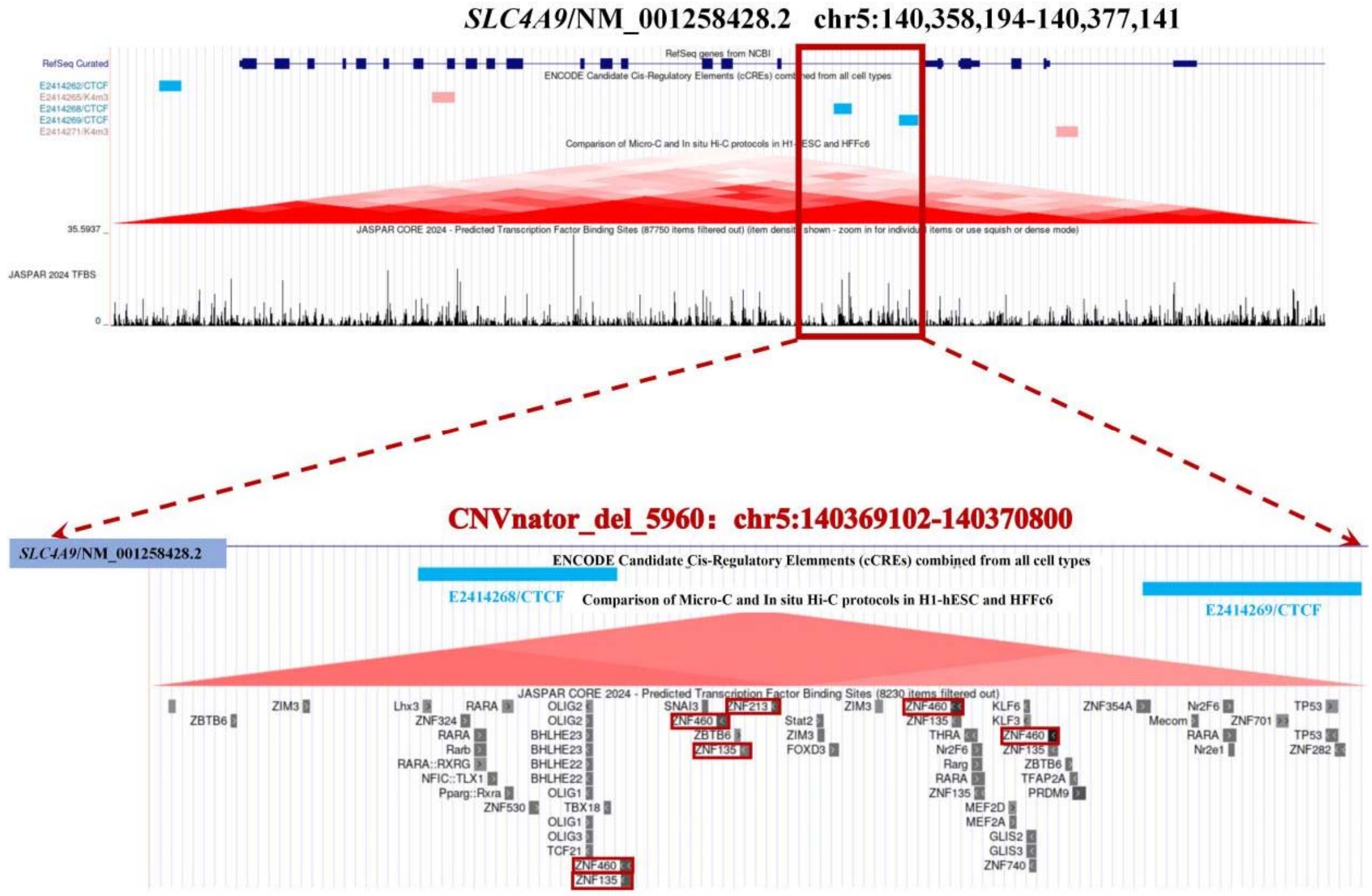

Supplementary Fig. 1 Analysis of CNV deletion region in human *SLC4A9*. The upper panel shows the bioinformatics analysis of ChIP-seq data from ENCODE database, micro-C chromatin structure and transcription factor binding site prediction of the *SLC4A9* genome. The red box area is the copy number variant (chr5:140369102-140370800, CNV deletion) area screened in the SS patient and is enlarged in the figure of lower panel. The CNV deletion region of *SLC4A9* has two CTCF (blue rectangle) occupancy positions. The transcription factors with high-affinity binding activity in the region analyzed were all zinc finger proteins such as *ZNF460, ZNF135, PRDM9* (red box marked) .

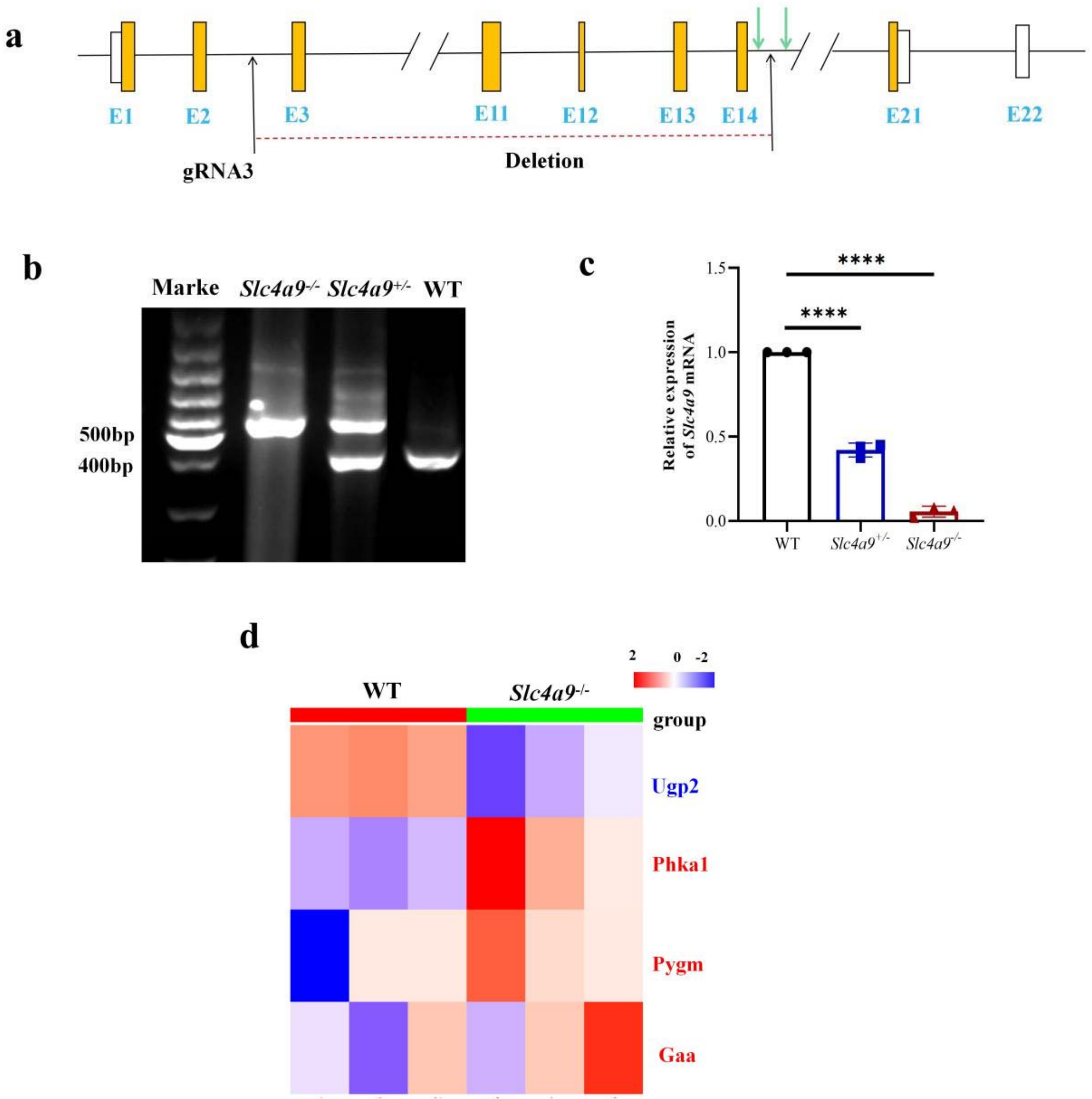

Supplementary Fig. 2 Generation and characterization of *Slc4a9* knockout mice. **a** Schematic representation of the *Slc4a9* knockout strategy, targeting the genomic region from upstream of exon 3 to downstream of exon 14. **b** PCR genotyping of tail DNA. Wild-type (WT) alleles yielded a 433-bp product, homozygous (*Slc4a9*$^{-/-}$) alleles produced a 560-bp product, and heterozygous (*Slc4a9*$^{+/-}$) samples displayed both bands. **c** RT-qPCR analysis of *SLC4A9* expression to verify gene knockout efficiency. Data are presented as mean ± SEM. Statistical significance was determined by t-test: **** $p < 0.00001$. **d** Heatmap of differentially expressed glycogen-related enzymes from SMG RNA-seq. Glycoprotein-degrading enzymes (*Ugp2*) were suppressed, while glycogen synthase (*Pygm, Gaa*, *Phka1*) were enhanced.

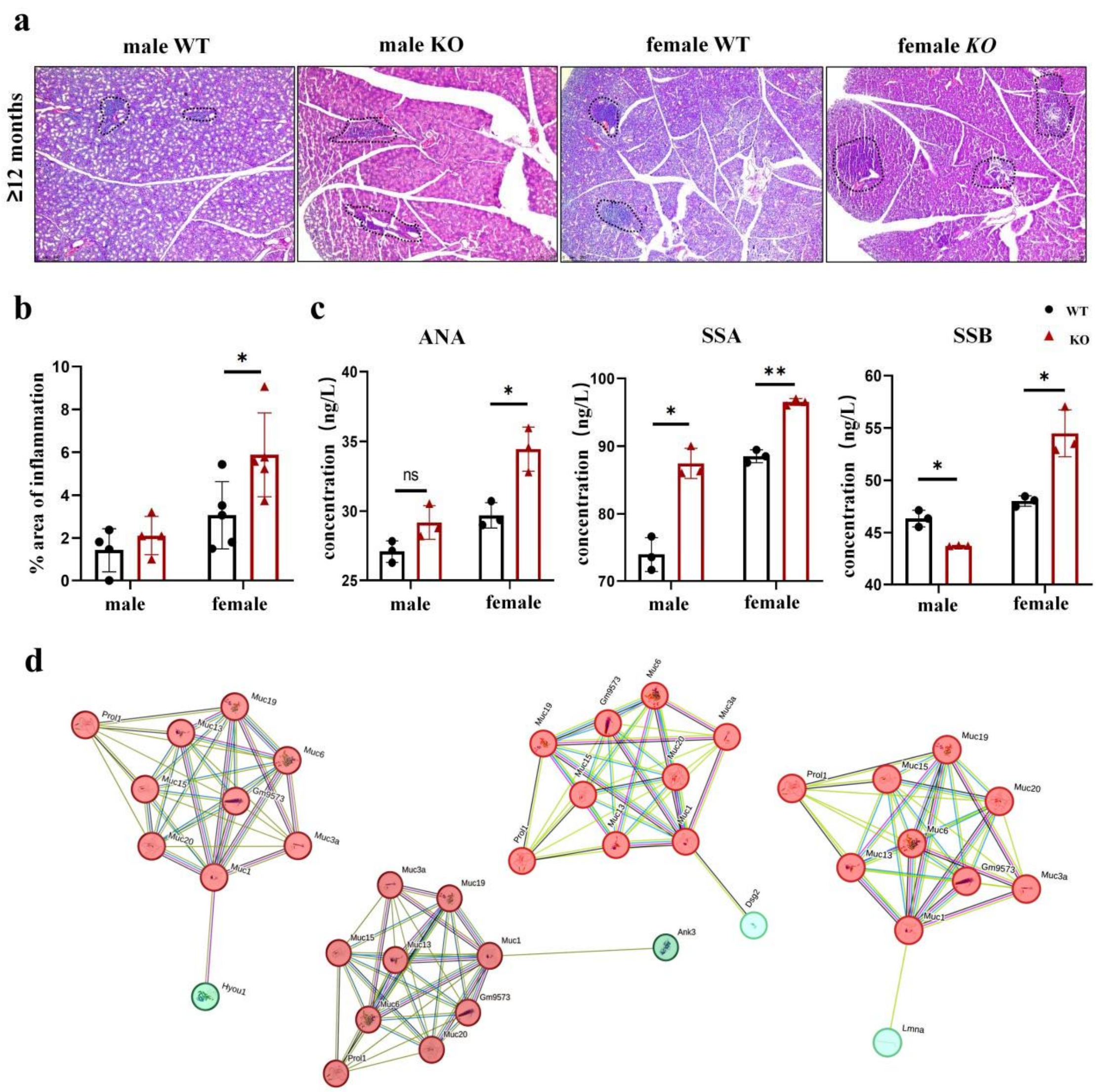

Supplementary Fig. 3 Age-related alterations and glycoprotein network changes in mouse salivary glands. **a** H&E staining of the submandibular gland. In older cohorts (≥ 12 months), both WT and KO mice showed some degree of infiltration, yet the infiltrates in KO mice were consistently larger in area and contained more focal lesions. **b** Quantification of inflammatory area. The area of lymphocytic infiltration was significantly larger in KO mice compared to age-matched WT controls, with a more pronounced increase in females. Data are presented as individual points. Statistical significance was determined by t-test: * $p < 0.05$. **c** Autoantibody titers in aged mice. Female KO mice (≥ 12 months) showed elevated levels of anti-ANA, anti-SSA/Ro, and anti-SSB/La autoantibodies relative to age-matched WT controls. Data are represented as individual points for each subject. Statistical significance was determined by t-test: * $p < 0.05$, ** $p < 0.01$. **d** Proteins interaction network of mucin family proteins and glycosylation-modified differential proteins. The differentially glycosylated proteins (green) in salivary glands interact with mucins (red). This network analysis reveals that the inclusion of proteins potentially affected in glycosylation (HYOU1, ANK3, DSG2, LMNA) and their interactions with mucins (e.g., HYOU1–MUC1) suggests potential roles in mucin function, modification, or localization.

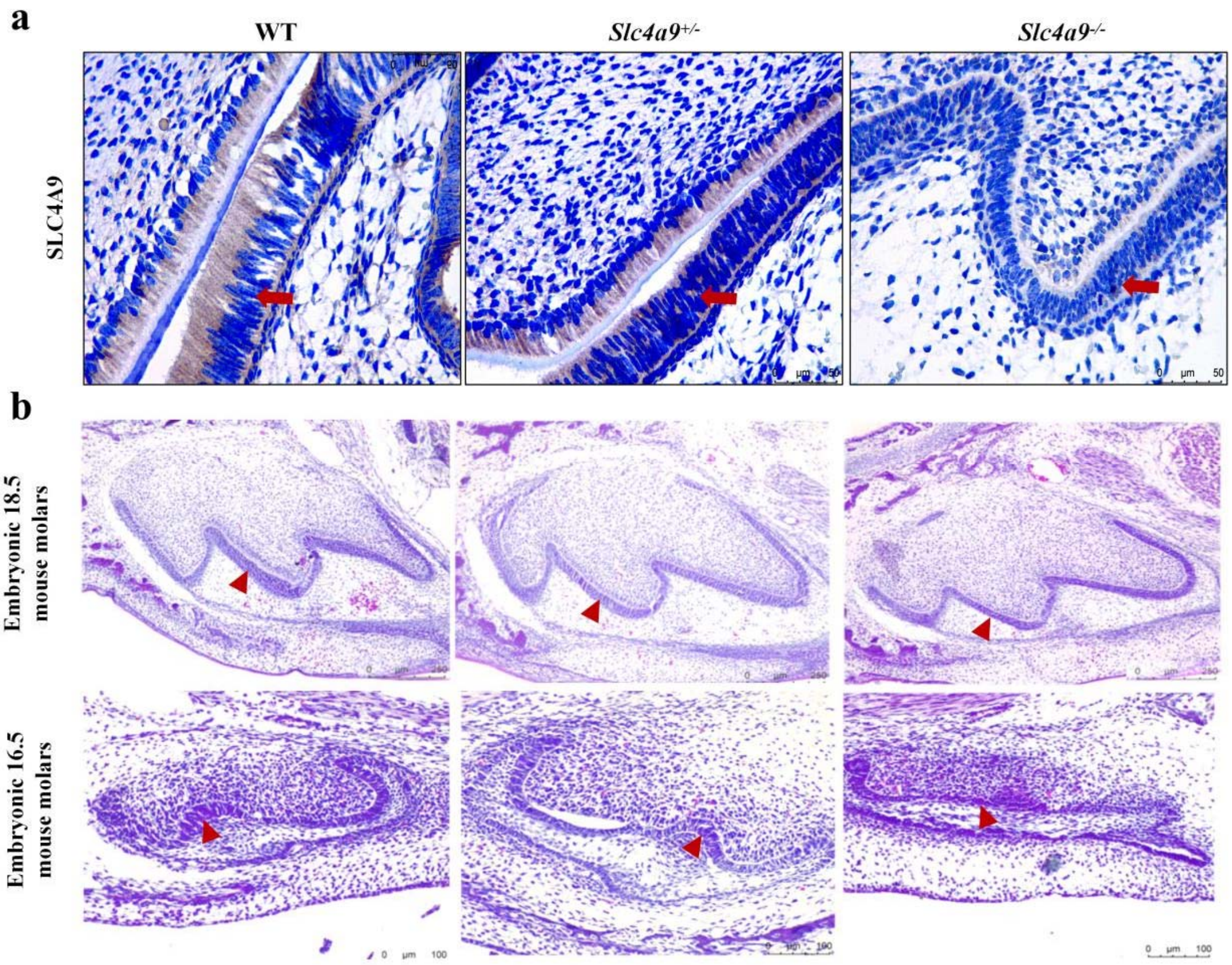

Supplementary Fig. 4 *Slc4a9* deficiency affects enamel development in mice. **a** Immunohistochemical staining detecting SLC4A9 expression in tooth germs. SLC4A9 is expressed in ameloblasts of wild-type mice, while its expression is downregulated in homozygous knockout mice. **b** Hematoxylin and eosin (H&E) staining of maxillary first molar germs from embryonic day 16.5 (E16.5) and E18.5 wild-type, *Slc4a9*$^{+/-}$ and *Slc4a9*$^{-/-}$ mice. Red arrows indicate ameloblasts

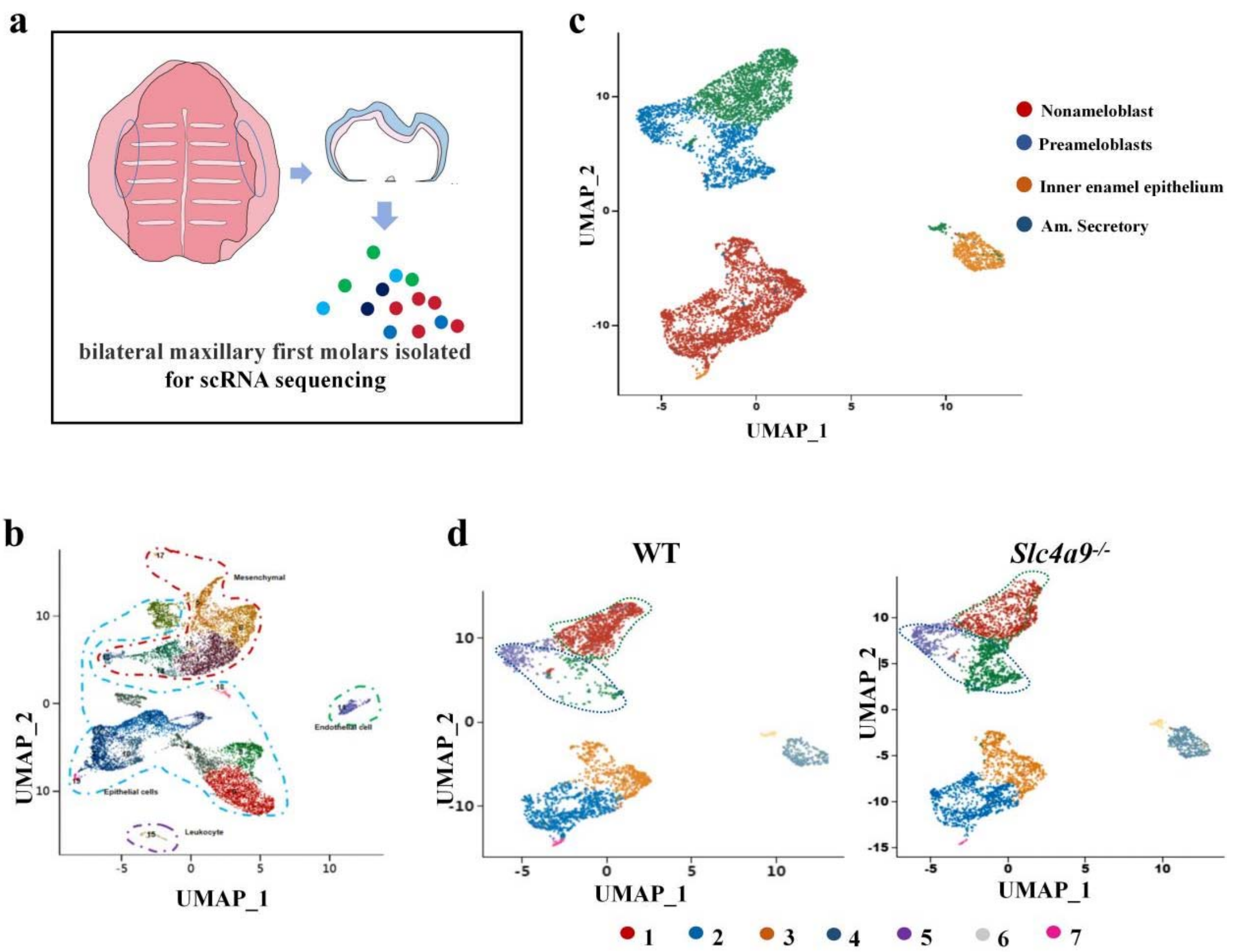

Supplementary Fig. 5 Single-cell sequencing of 1-day postnatal mouse maxillary first molars. **a** A schematic depicting view of the region isolated for single-cell RNA sequencing (scRNAseq). **b** UMAP-based projection of the entire scRNA-seq data set, with larger groups, based on gene expression profiles, outlined. The epithelial cells, outlined in red blue, were subset and reclustered in the following panels. **c** UMAP-based projection of the reclustered epithelial cells reveals 4 distinct clusters. Individual clusters were identified based on gene expression profiles. **d** UMAP-based projections, as shown in panel **c**, but split by genotype. The control UMAP is on the left; the *Slc4a9* knockout UMAP is on the right. Clusters 1, 4 and 5 are circled in both UMAPs.

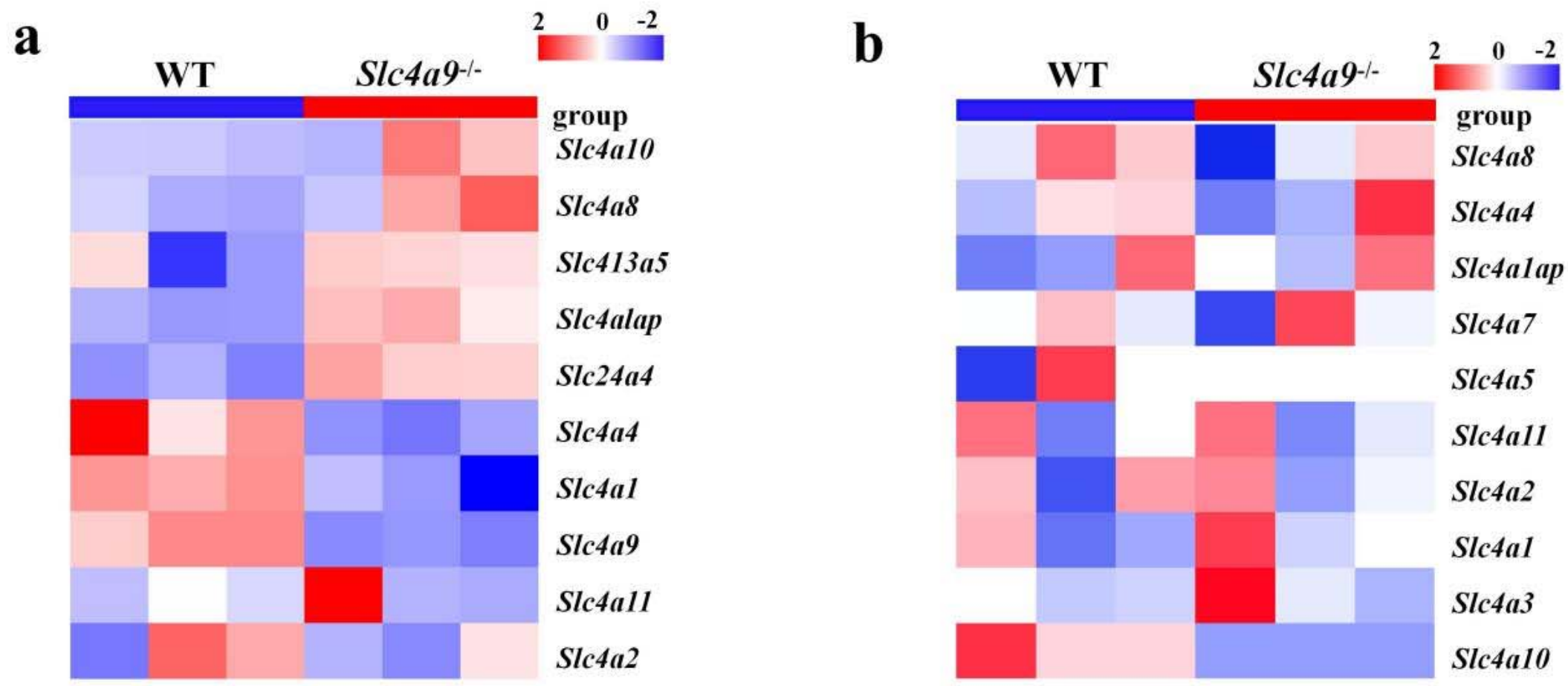

Supplementary Fig. 6 Heatmap of SLC4 family members in tooth germ tissue and submandibular salivary glands. **a** Heatmap of SLC4 family members in tooth germ tissue from wild type and *Slc4a9*$^{-/-}$ mice through transcriptome sequencing. The expression of *Slc4a9* was significantly downregulated in homozygous mice compared with wild-type, while the expression of *Slc4a10*, *Slc4a8* etc. was slightly upregulated. **b** Heatmap of SLC4 family members in submandibular salivary glands from wild type and *Slc4a9*$^{-/-}$ mice through transcriptome sequencing. There is no significant difference in the expression of *Slc4a2* between wild-type and homozygous mice.

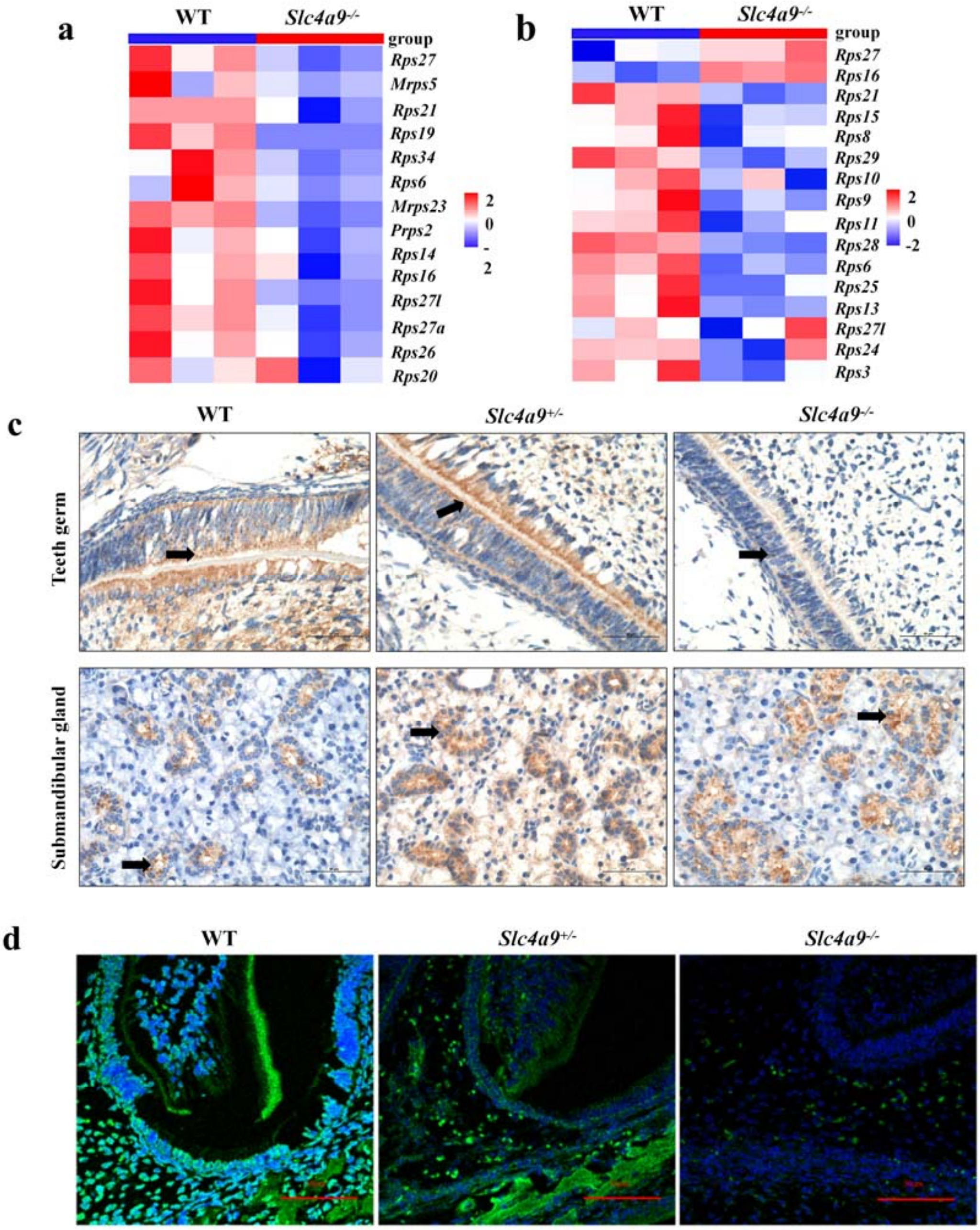

Supplementary Fig. 7 Ribosomal stress and apoptotic priming in *Slc4a9*-deficient secretory epithelia. **a-b** Heatmap of tooth germ tissue and submandibular salivary glands from wild-type and *Slc4a9*$^{-/-}$ mice through transcriptome sequencing. A wide range of ribosomal genes have differences between wild type and *Slc4a9*$^{-/-}$ mice, among which the expression of *Rps27* is the most significantly increased in both tooth germ tissues (A) and salivary glands (B). **c** The expression of RPS27 in tooth germ and submandibular salivary gland paraffin sections detected by immunohistochemical staining. Positive signal: brown. Blacked arrows indicates ameloblast and duct cells respectively. **d** TUNEL staining was used to observe the apoptosis of tooth germ tissues. Green fluorescence indicates cell apoptosis, the greener indicates the higher degree of apoptosis, and blue indicates the cell nucleus.

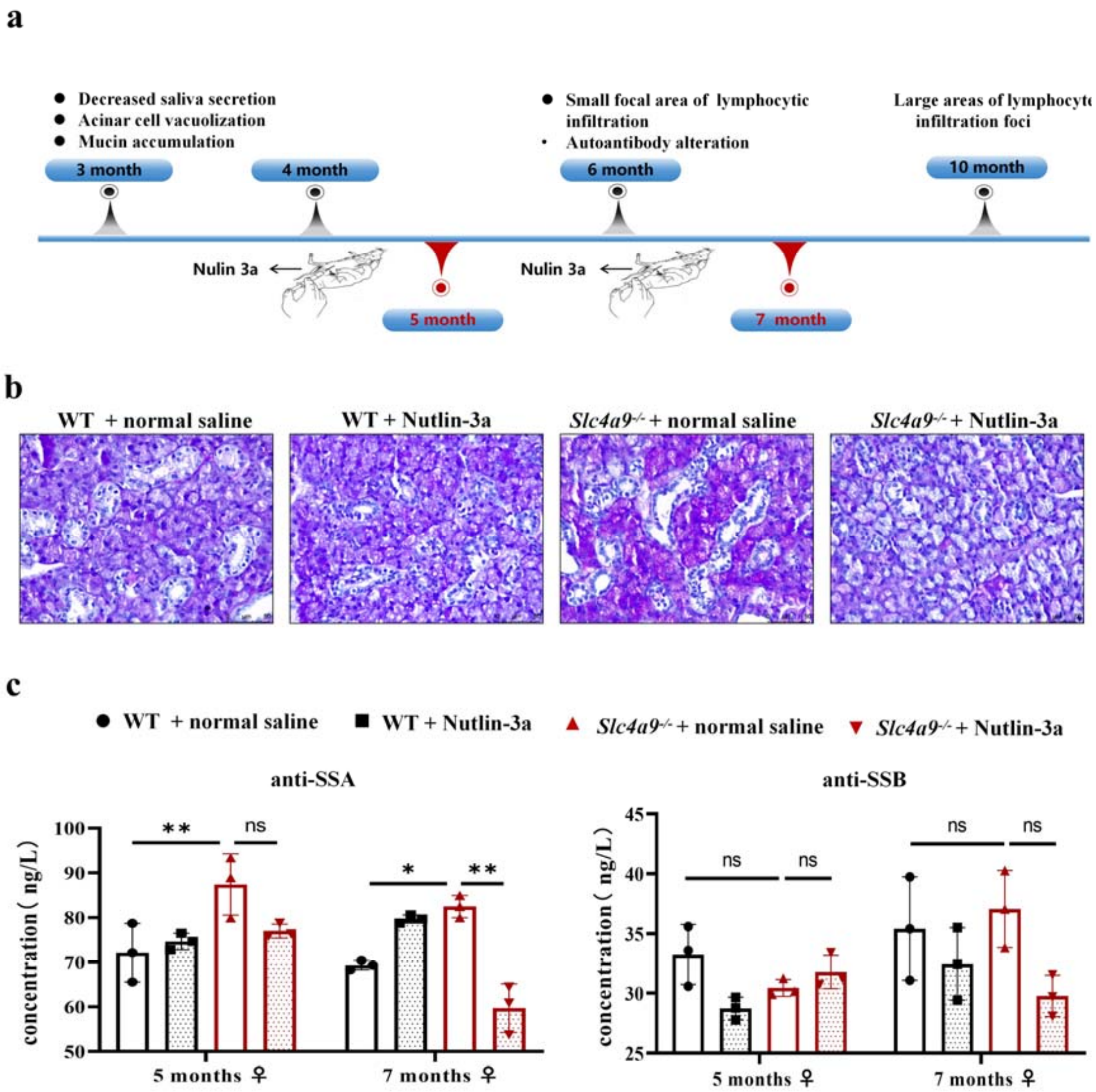

Supplementary Fig. 8 Nutlin-3a restores salivary gland inflammatory phenotype in *Slc4a9*$^{-/-}$ mice by reversing RPS27-MDM2-p53 axis dysfunction. **a** The timeline of Sjögren's syndrome phenotype manifestation in *Slc4a9*$^{-/-}$ mice. *Slc4a9*$^{-/-}$ and WT mice in 4 months and 6 months treated with Nutlin-3a (20 mg/kg, every 48 hours for 1 month) or vehicle-treated controls. Phenotype of the submandibular glands in 5 months and 7 months were observed. **b** Periodic acid-Schiff staining of salivary gland sections from 5-month-old mice. The group of Nutlin-3a treatment restored mucin expression of *Slc4a9*$^{-/-}$ mice to near-normal levels compared with vehicle-treated controls. **c** Female autoantibody profiles. Serum autoantibody levels across four groups showed a significant reduction in autoantibody production in Nutlin-3a-treated mice. Data are represented as individual points for each mouse. Statistical significance was determined by t-test: * $p < 0.05$, ** $p < 0.01$.

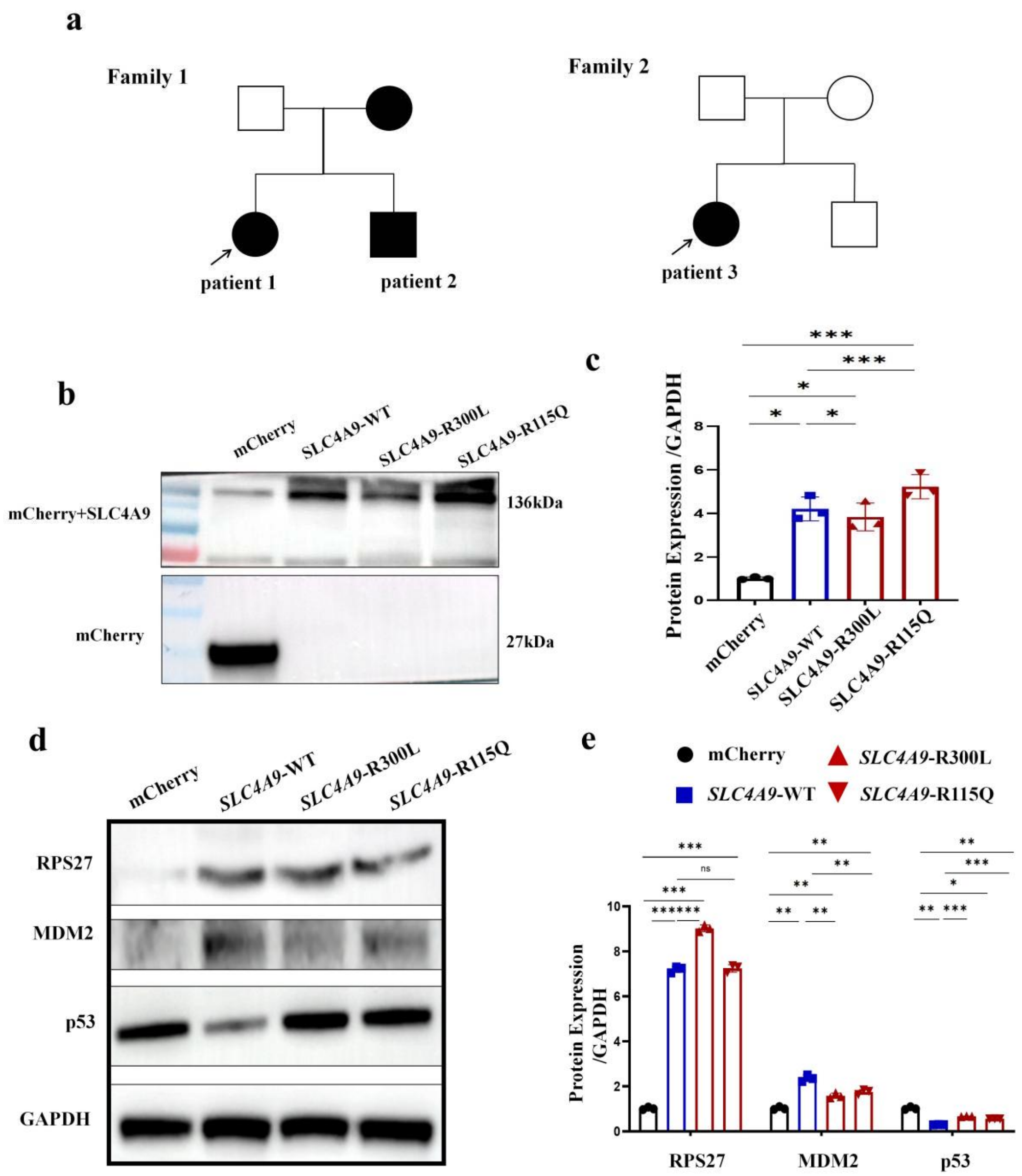

Supplementary Fig. 9 Establishment of overexpression *SLC4A9* mutant cell lines. **a** Pedigree of the family 1. Males are marked as squares and females as circles. Filled symbols designate affected individuals; open symbols indicate those not affected. The black arrow points to the proband. **b** Western blotting analysis of stably overexpression *SLC4A9*-WT, *SLC4A9*-R300L and *SLC4A9*-R115Q mutants in LS8 cells. **c** Quantitative analysis of western blot. Mean values ± SEM are shown, n = 3 (*$p < 0.05$, **$p < 0.01$, ***$p < 0.001$, **** $p < 0.0001$, t -test). **d-e** Activation of stress signaling. d Western blot analysis shows RPS27 upregulation and p53-MDM2 axis activation in SLC4A9-mutant LS8 cells. e Quantitative analysis of western blot. Mean values ± SEM are shown, n = 3 (* $p < 0.05$, ** $p < 0.01$, *** $p < 0.001$, **** $p < 0.0001$, t-test).

Supplementary Table 1 Sjögren's Syndrome diagnostic score based on combined European-American consensus

Case	Age	Gender	Criteria iterms (score)
1	30	female	**Score = 8**<br>(1) Labial salivary gland with focal lymphocytic sialadenitis and focus score of ≧1 foci/4 mm2: 3 points<br>(2) Anti-SSA/Ro positive: 3 points<br>(3) Schirmer's test ≦5 mm/5 minutes in at least 1 eye: 1 point<br>(4) Unstimulated whole saliva flow rate ≦0.1 ml/minute: 1 point
2	47	female	**Score = 4**<br>(1) Labial salivary gland with focal lymphocytic sialadenitis and focus score of ≧1 foci/4 mm2: 3 points<br>(3) Schirmer's test ≦5 mm/5 minutes in at least 1 eye: 1 point
3	32	female	**Score = 7**<br>(1) Labial salivary gland with focal lymphocytic sialadenitis and focus score of ≧1 foci/4 mm2: 3 points<br>(2) Anti-SSA/Ro positive: 3 points<br>(3) Unstimulated whole saliva flow rate ≦0.1 ml/minute: 1 point
4	44	female	**Score = 4**<br>(1) Labial salivary gland with focal lymphocytic sialadenitis and focus score of ≧1 foci/4 mm2: 3 points<br>(2) Unstimulated whole saliva flow rate ≦0.1 ml/minute: 1 point
5	47	female	**Score = 6**<br>(1) Labial salivary gland with focal lymphocytic sialadenitis and focus score of ≧1 foci/4 mm2: 3 points<br>(2) Anti-SSA/Ro positive: 3 points

Supplementary Table 2 Mass spectrometric data for selected 40 O-glycans obtained from mouse submandibular glands

Protein_Position	WT	*Slc4a9*$^{-/-}$	FC	pValue	Sig
DYHC1_S4248	36.96896291	0.108701901	340.0948729	0.001776508	-1
SMC1A_T1195	0.08517023	2.920638472	0.029161511	0.023254909	1
VIGLN_T1102	17.91975776	0.236512987	75.76648406	0.016197637	-1
ANK3_T1088	0.112667774	2.116580891	0.053231027	0.047574199	1
SMC1A_T1041	0.103975122	1.981651376	0.052468927	0.046814589	1
EDC4_S937	0.155180891	8.488849109	0.018280557	0.012528021	1
AP2B1_S933	23.30608125	0.166382988	140.0748952	0.00700486	-1
TBCD4_S748	4.755791067	0.121537647	39.13018911	0.036081201	-1
AT2A3_S722	5.622003533	0.087687682	64.11394844	0.020025846	-1
LMNA_S611	0.054300166	1.991944507	0.027259879	0.021341094	1
HYOU1_T598	0.14517977	7.724323115	0.018795145	0.013017519	1
RRBP1_S587	0.099620336	2.157231297	0.04617972	0.040505318	1
MGT4A_T477	0.076932484	1.492951443	0.051530466	0.045877632	1
LAMC3_S466	0.220660181	7.134705751	0.03092772	0.025039792	1
2ABA_S409	0.054121894	15.63064071	0.003462551	0.000872522	1
LMAN1_S393	3.287055261	0.107056716	30.70386837	0.047184054	-1
LMAN1_T385	0.099622497	2.217796673	0.044919581	0.039233516	1
DSG2_S368	0.063486335	1.178488849	0.053870968	0.04821115	1
DNJA1_T355	0.056316897	1.120011934	0.050282408	0.044628989	1
ASM3A_S300	0.162702141	136.6599538	0.001190562	0.000107144	1
K1B26_T244	2.631188494	0.086831794	30.30213226	0.047857935	-1
UBP14_S222	7.104718647	0.125151682	56.76886387	0.023276303	-1
LAMA2_T196	3.620640929	0.12117715	29.87890825	0.04858608	-1
AFG32_S164	0.090673192	14.73707765	0.006152725	0.002378798	1
K1B26_T161	0.161139141	3.954053852	0.040752895	0.035014668	1
1433Z_S145	34.57380772	0.052714074	655.8744709	0.000567619	-1
GSTO1_S139	10.58894777	0.116836194	90.63071455	0.012805604	-1
NGF_S134	0.11694388	2.124487208	0.055045697	0.049378199	1
MIC60_S128	4.797022458	0.143760477	33.36815857	0.043100423	-1
NEUM_S122	0.11201978	2.113970314	0.052990233	0.047334317	1
PSA3_S97	13.83951552	0.239288371	57.83614061	0.022751655	-1
K1KB9_S95	0.097725495	4.222570299	0.023143604	0.017241962	1
MCM3_T90	0.159704271	6.366748713	0.025084117	0.019165405	1
CTBL1_T88	4.633711834	0.069844264	66.34348543	0.019185117	-1
EF1B_T88	0.129454873	4.834116506	0.026779428	0.020859211	1
NGF_T71	4.001160737	0.135854067	29.45190245	0.049340402	-1
GRPE1_S55	5.285894524	0.08118468	65.10950768	0.019643086	-1
ARGI1_T43	4.648145344	0.146990851	31.62200422	0.045704013	-1
SRBS2_S43	6.128690386	0.103062372	59.46583741	0.021987471	-1
6PGD_T35	0.095802628	2.809577087	0.034098594	0.028255347	1
